# AI-Driven Wearable Bioelectronics in Digital Healthcare

**DOI:** 10.3390/bios15070410

**Published:** 2025-06-26

**Authors:** Guangqi Huang, Xiaofeng Chen, Caizhi Liao

**Affiliations:** 1 Department of Bioelectronics, Faculty of Biomedical Engineering, Shenzhen University of Advanced Technology, Shenzhen 518055, China; gqhuang.pku@outlook.com; 2 Division of Electrical Engineering, Department of Engineering, Cambridge University, Cambridge CB2 1TN, UK; 3 Department of Chemistry, The University of Texas at Austin, Austin, TX 78712, USA; x.chen@utexas.edu

**Keywords:** artificial intelligence (AI), wearable bioelectronics, digital healthcare, disease diagnosis, healthcare monitoring

## Abstract

The integration of artificial intelligence (AI) with wearable bioelectronics is revolutionizing digital healthcare by enabling proactive, personalized, and data-driven medical solutions. These advanced devices, equipped with multimodal sensors and AI-powered analytics, facilitate real-time monitoring of physiological and biochemical parameters—such as cardiac activity, glucose levels, and biomarkers—allowing for early disease detection, chronic condition management, and precision therapeutics. By shifting healthcare from reactive to preventive paradigms, AI-driven wearables address critical challenges, including rising chronic disease burdens, aging populations, and healthcare accessibility gaps. However, their widespread adoption faces technical, ethical, and regulatory hurdles, such as data interoperability, privacy concerns, algorithmic bias, and the need for robust clinical validation. This review comprehensively examines the current state of AI-enhanced wearable bioelectronics, covering (1) foundational technologies in sensor design, AI algorithms, and energy-efficient hardware; (2) applications in continuous health monitoring, diagnostics, and personalized interventions; (3) key challenges in scalability, security, and regulatory compliance; and (4) future directions involving 5G, the IoT, and global standardization efforts. We highlight how these technologies could democratize healthcare through remote patient monitoring and resource optimization while emphasizing the imperative of interdisciplinary collaboration to ensure equitable, secure, and clinically impactful deployment. By synthesizing advancements and critical gaps, this review aims to guide researchers, clinicians, and policymakers toward responsible innovation in the next generation of digital healthcare.

## 1. Introduction

The rapid convergence of artificial intelligence (AI) and wearable bioelectronics is ushering in a transformative era in digital healthcare, fundamentally redefining how healthcare is delivered [1,2,3]. Traditionally, healthcare has relied on reactive and generalized approaches, where patients seek medical attention only after symptoms arise, and treatments are often standardized rather than tailored to individual needs. However, the synergy between AI and wearable bioelectronics is shifting this paradigm toward proactive, personalized, and data-driven solutions [4,5,6]. Wearable bioelectronics, equipped with advanced sensors and AI algorithms, enable continuous, real-time monitoring of physiological and biochemical parameters, such as heart rate, blood pressure, glucose levels, and even biomarkers in sweat or interstitial fluid [7,8,9,10,11]. This capability offers unprecedented opportunities for early disease detection, chronic disease management, and personalized therapeutic interventions. For example, wearable devices can detect irregular heart rhythms before they lead to a heart attack or monitor blood sugar levels in real time to prevent diabetic complications [12,13,14]. The integration of AI into these devices enhances their capabilities by enabling sophisticated data analysis, pattern recognition, and predictive modeling. This allows for the extraction of actionable insights from vast amounts of health data, facilitating timely interventions and significantly improving patient outcomes [15,16].

Moreover, AI-driven wearable bioelectronics are poised to address some of the most pressing challenges in modern healthcare [17,18,19]. The growing burden of chronic diseases, such as diabetes (422 million cases of diabetes worldwide (WHO, 2023)), cardiovascular diseases (leading to 17.9 million lives lost annually), and respiratory conditions has placed immense strain on healthcare systems worldwide [20,21]. At the same time, aging populations (projected doubling of the aged population (60+ years) by 2050) are increasing the demand for long-term care and monitoring. Wearable bioelectronics, powered by AI, offer cost-effective and scalable solutions to these challenges by enabling continuous health monitoring outside traditional clinical settings [1,22,23,24]. For instance, remote patient monitoring using wearable devices can reduce hospital readmissions and optimize resource allocation, easing the burden on healthcare providers [20,25]. These technologies also empower individuals to take a proactive role in managing their health, fostering a shift from episodic care to continuous wellness management. By democratizing access to healthcare, AI-driven wearable bioelectronics have the potential to make healthcare more accessible, affordable, and personalized, particularly for underserved populations and those in remote areas [17,18,26,27,28].

While predictive analytics represent a key application, AI enhances wearable bioelectronics through three critical dimensions: (1) real-time anomaly detection via edge-processed multimodal data streams, enabling sub-second identification of acute events like cardiac arrhythmias; (2) adaptive therapeutic interventions through closed-loop systems that dynamically adjust neurostimulation parameters or drug delivery; and (3) context-aware signal interpretation that discriminates biomarkers from artifacts using behavioral and environmental data. These capabilities are enabled by lightweight neural networks (<5 mW power consumption) that balance clinical-grade accuracy with stringent energy constraints—a technological paradigm explored in depth in Section 3.

The realization of continuous, AI-driven health monitoring through wearables faces two fundamental technological hurdles: sustainable power delivery and reliable data transmission [29,30,31]. Power management remains challenging due to the conflicting demands of sophisticated AI processing and extended battery life, necessitating energy-efficient edge computing architectures that optimize operations per watt. Simultaneously, real-time functionality requires robust wireless protocols like ultra-wideband and Bluetooth LE 5.2 that ensure secure, low-latency data transmission within strict energy budgets. Emerging solutions integrate novel power harvesting (kinetic, thermal, and RF energy scavenging) with adaptive AI algorithms that dynamically adjust computational complexity based on available power [31]. These co-design approaches crucially balance clinical utility with practical operational constraints, enabling intelligent wearables that maintain both analytical precision and weeks-long operation between charges—a prerequisite for meaningful healthcare applications.

**Figure 1:**
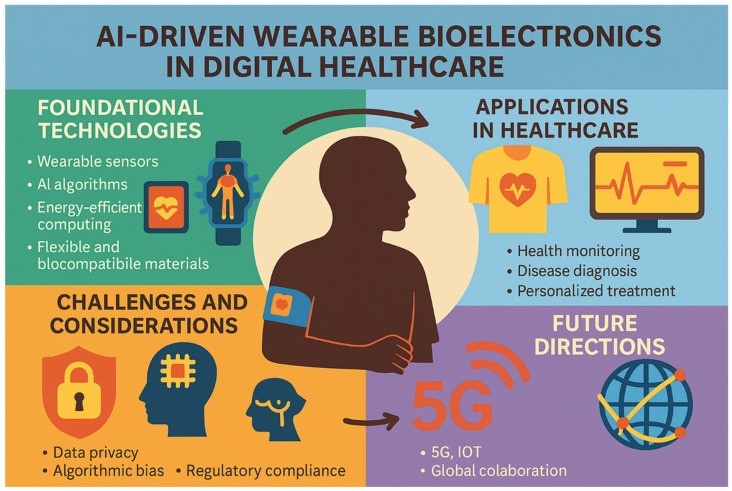
AI-driven wearable bioelectronics and their broad applications in digital healthcare.

This review paper aims to provide a comprehensive overview of the current state, applications, challenges, and prospects of AI-driven wearable bioelectronics in digital healthcare, as summarized in Figure 1. The paper is structured to guide readers through the key aspects of this rapidly evolving field. Section 2 delves into the fundamental technologies underpinning these devices, including advancements in wearable sensors, AI algorithms, and energy-efficient computing. It explores how innovations in materials science, such as flexible and biocompatible materials, have enabled the development of wearable devices that are comfortable, durable, and capable of continuous operation. Section 3 explores the diverse applications of AI-driven wearable bioelectronics, focusing on health monitoring, diagnosis, and personalized treatment. It highlights how these devices are being used to monitor vital signs, detect diseases early, and deliver tailored therapeutic interventions. Section 4 and Section 5 discuss the technical, ethical, and regulatory challenges that must be addressed to ensure the safe and effective deployment of these technologies. Issues such as data privacy, algorithmic bias, and regulatory compliance are critical to building trust and ensuring the widespread adoption of wearable bioelectronics. Finally, Section 5 outlines future directions and opportunities for innovation in this field, including the integration of emerging technologies like 5G, the IoT, and quantum computing, as well as the potential for global collaboration to accelerate progress.

The significance of AI-driven wearable bioelectronics lies in their potential to revolutionize healthcare by making it more proactive, personalized, and accessible. By enabling continuous health monitoring outside traditional clinical settings, these technologies empower individuals to take control of their health and well-being [2,32]. For healthcare providers, wearable bioelectronics offer valuable tools for remote patient monitoring, reducing the need for frequent hospital visits and enabling more efficient resource allocation [33,34]. As the global healthcare landscape continues to evolve, AI-driven wearable bioelectronics are set to play a pivotal role in shaping the future of digital healthcare [3,35]. This review seeks to synthesize the latest research, highlight key advancements, and identify critical gaps in knowledge, offering a holistic perspective on the transformative potential of these technologies. By doing so, it aims to inform researchers, clinicians, policymakers, and industry stakeholders about the opportunities and challenges associated with AI-driven wearable bioelectronics, ultimately contributing to their responsible and impactful integration into healthcare systems worldwide. Through this comprehensive exploration, the review underscores the importance of interdisciplinary collaboration and continued innovation to fully harness the benefits of AI-driven wearable bioelectronics in improving health outcomes and enhancing quality of life.

## 2. Wearable Bioelectronics

Wearable bioelectronics serve as the physical interface between the human body and digital healthcare systems. These devices are engineered to monitor physiological and biochemical signals continuously and non-invasively, providing critical data for health assessment, disease management, and personalized interventions [36]. Key aspects of wearable bioelectronics include their design, functionality, and the technologies that enable their operation. Below, we explore the core components and advancements in this field.

The core functionality of wearable bioelectronics relies on three integrated components, each with distinct performance metrics that have evolved significantly. The bioreceptor component has advanced from early enzyme-based systems (e.g., glucose oxidase with K~m~ = 10–30 mM) to synthetic molecularly imprinted polymers (MIPs) achieving dissociation constants (K~d~) < 1 nM while maintaining 90% specificity in complex media. For the transducer element, electrochemical platforms have seen a 1000× sensitivity improvement (from μA/mM to nA/pM ranges) through nanostructured materials like laser-scribed graphene (charge transfer efficiency > 95%), while optical transducers now achieve attomolar detection limits via plasmonic enhancement (EF > 10^8^) [37]. The processor unit has undergone revolutionary changes, with edge AI implementations reducing latency from 500 ms (cloud-dependent) to <20 ms (on-device TinyML models) while cutting power consumption from 100 mW to <5 mW per analysis cycle through spiking neural network architectures [38].

The synergistic optimization of these components follows clear trends: (1) Bioreceptor–transducer pairs now achieve 99% signal fidelity (vs. 70% in 2010) through matched impedance designs (e.g., PEDOT:PSS interfaces reducing charge transfer resistance from 10^5^ to 10^2^ Ω) [39]; (2) Modern systems integrate all three components on flexible substrates with <5% performance degradation after 10,000 bending cycles (vs. complete failure at 100 cycles in early designs); (3) End-to-end system efficiency (defined as [sensitivity × speed]/power) has improved 10^6^-fold since 2010, enabling continuous multi-analyte monitoring at <1 mW power budgets [17]. These metrics are benchmarked against clinical requirements, demonstrating how component-level innovations collectively enable detection of biomarkers at <1 pg/mL concentrations with <3% CV—performance unattainable with any single-component optimization.

### 2.1. Overview of Wearable Sensors and Bioelectronics

#### 2.1.1. Biosensors

**Figure 2:**
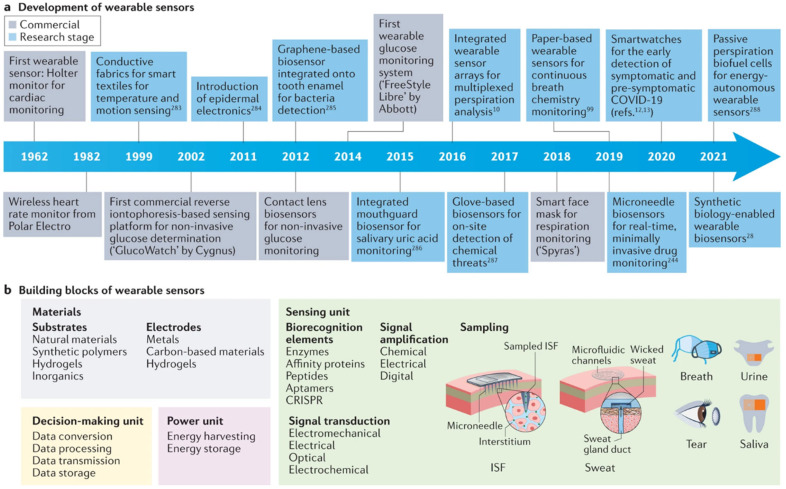
A timeline of key milestones in wearable sensor development (**a**), along with a summary of their core components (**b**). Reproduced with permission [60]. Copyright 2022, Springer Nature Limited.

Biosensors are a pivotal technology in wearable bioelectronics, enabling the detection and measurement of key biomarkers such as glucose, lactate, cortisol, and electrolytes in bodily fluids like sweat, saliva, and interstitial fluid [38,40,41,42,43] (see Figure 2). These devices consist of three main components: a bioreceptor that interacts with the target biomarker, a transducer that converts this interaction into a measurable signal, and a signal processor that interprets the data [44]. For example, glucose biosensors use enzymes like glucose oxidase to detect glucose levels, providing real-time feedback for diabetes management [45,46]. By offering non-invasive continuous monitoring, biosensors empower individuals to track their health in real time, facilitating early detection of abnormalities and personalized interventions.

The evolution of wearable sensors can be systematically analyzed through four fundamental performance metrics that have driven iterative improvements across generations of devices.

**Response Time**: Early-generation sensors (pre-2010) typically exhibited response times >30 s due to reliance on slow diffusion-limited processes. The shift to nanostructured materials (e.g., vertically aligned carbon nanotubes) and microfluidic designs reduced this to <5 s by enabling direct analyte access to active sites. Recent work on field-effect transistor architectures has achieved sub-second response (0.2–0.5 s) through gating effect optimization, critical for real-time monitoring in acute care scenarios [47]. However, these gains often require trade-offs with power consumption—an ongoing challenge addressed through adaptive sampling algorithms.

**Sensitivity**: Sensitivity improvements have followed three parallel pathways: (a) *Material innovations* (e.g., transition from bulk metals to graphene/2D materials yielding 100–1000× signal amplification), (b) *Surface engineering* (hierarchical nanostructures increasing effective sensing area), and (c) *Signal processing* (machine learning-based noise reduction enabling reliable pM-level detection) [48]. Notably, plasmonic sensors now achieve attomolar sensitivity for specific biomarkers, though with selectivity constraints in complex media. The development of amplification strategies like catalytic hairpin assembly has pushed detection limits while maintaining rapid response.

**Stability**: Long-term stability has progressed from hours (early enzymatic sensors) to months of continuous operation through the following:

*Interface engineering*: Anti-fouling coatings (e.g., zwitterionic polymers) reducing biofouling-induced drift to <2%/day.

*Self-calibration architectures*: Reference-integrated designs compensating for environmental variability.

*Materials selection*: Transition from noble metals to stable conductive polymers (PEDOT:PSS) resisting corrosion.

Recent studies demonstrate 6-month stability in implantable glucose sensors via multilayer encapsulation, though challenges remain in extreme conditions (high humidity, mechanical stress).

**Selectivity**: The selectivity paradigm has evolved from *physical separation* (early membranes), *biorecognition elements* (antibodies/aptamers), and *multi-parametric sensing* (cross-reactive arrays) to *AI-driven deconvolution* (neural networks resolving overlapping signals). Current state-of-the-art methods combine molecularly imprinted polymers (specificity > 90%) with multi-modal detection (e.g., simultaneous electrochemical/optical readouts), achieving discrimination between structurally similar molecules (e.g., dopamine vs. uric acid) [49]. Machine learning further enhances specificity by learning individual baselines and interference patterns.

**Trade-off Analysis**: Table 1 illustrates how sensor generations have balanced these metrics:

1st-gen: Prioritized selectivity (antibody-based) at expense of response time.

2nd-gen: Optimized sensitivity (nanomaterials) with moderate stability.

3rd-gen: Achieved balanced performance through system-level integration.

This metrics-focused analysis provides clear benchmarks for sensor selection and identifies remaining challenges (e.g., simultaneous optimization of rapid response and long-term stability) as key research frontiers, see Table 1.

**Table 1:** Performance matrix of different generations of biosensors.

Generation	Response Time	Sensitivity	Stability	Selectivity
**2000–2010**	>30 s	μM–mM	Hours/days	Antibody dependent
**2010–2020**	2–10 s	nM–μM	Weeks	Aptamer/MIP-based
**2020-present**	<1 s	pM–nM	Months	AI-enhanced multimodal

The integration of biosensors into wearable devices such as patches, smartwatches, and smart clothing has revolutionized digital healthcare [50,51]. Wearable patches equipped with biosensors can monitor biomarkers like glucose or electrolytes through sweat, while advanced smartwatches use optical sensors to analyze sweat composition during physical activity. Smart clothing with embedded biosensors offers a seamless way to monitor stress hormones like cortisol or hydration status. These wearable platforms provide portability, convenience, and real-time feedback, making them ideal for everyday health tracking [52,53]. However, challenges such as ensuring accuracy across varying conditions, improving long-term stability, and achieving miniaturization and power efficiency must be addressed to enhance their performance and adoption.

While multiplexed detection offers significant advantages for comprehensive health monitoring, it introduces critical technical challenges: (1) Cross-sensitivity between structurally similar biomarkers (e.g., glucose vs. lactate in electrochemical sensors) can compromise specificity; (2) Signal interference (optical/electrical crosstalk) degrades accuracy in multi-analyte systems; and (3) Scalability limits emerge as power/computational demands grow exponentially with added sensing modalities. These challenges motivate the development of advanced solutions discussed in Section 4, including nanomaterial-based selective interfaces and machine learning-assisted signal decoupling approaches [54,55].

Looking ahead, biosensors hold immense potential to transform healthcare by enabling proactive and personalized health management. Advances in materials science, fabrication techniques, and AI-driven data analysis are expected to improve the accuracy, reliability, and multiplexing capabilities of biosensors, allowing for the simultaneous monitoring of multiple biomarkers [48,56]. As these technologies evolve, biosensors will play an increasingly critical role in digital healthcare, empowering individuals to take control of their health and providing healthcare professionals with valuable tools for remote monitoring and timely interventions. The future of biosensors lies in their ability to seamlessly integrate into wearable devices, making continuous health monitoring accessible, affordable, and effective for all [57,58,59].

#### 2.1.2. Smartwatches and Fitness Trackers

Smartwatches and fitness trackers have become ubiquitous tools for general health monitoring, offering users the ability to track a wide range of metrics such as heart rate, physical activity, sleep patterns, and blood oxygen levels (SpO2) [61,62,63]. These devices leverage advanced sensors, including optical heart rate monitors, accelerometers, gyroscopes, and pulse oximeters, to collect data on various aspects of an individual’s health and fitness [64]. By providing real-time feedback and long-term trends, smartwatches and fitness trackers empower users to make informed decisions about their lifestyle, exercise routines, and overall well-being. Their user-friendly interfaces, combined with seamless integration with smartphones and health apps, have made them popular among both fitness enthusiasts and individuals seeking to improve their health [65,66,67].

One of the key features of smartwatches and fitness trackers is their ability to monitor cardiovascular health through continuous heart rate tracking and SpO2 measurement [64,68,69]. Optical heart rate sensors use photoplethysmography (PPG) to detect blood flow changes, enabling the detection of resting heart rate, exercise intensity, and even irregular heart rhythms. SpO2 sensors, which measure blood oxygen saturation, provide insights into respiratory health and can alert users to potential issues such as sleep apnea or low oxygen levels during high-altitude activities. Additionally, these devices track physical activity through step counting, distance traveled, and calories burned, encouraging users to stay active and meet daily fitness goals. Sleep tracking features analyze sleep duration and quality, offering personalized recommendations to improve rest and recovery [70,71].

Despite their widespread adoption, smartwatches and fitness trackers face challenges related to accuracy, data interpretation, and user engagement [72,73]. While these devices provide valuable insights, their measurements may not always match the precision of clinical-grade equipment, particularly for metrics like SpO2 and sleep stages [74]. Ensuring data accuracy across diverse populations and activity levels remains an ongoing area of research. Furthermore, the sheer volume of data generated by these devices can overwhelm users, highlighting the need for intuitive interfaces and actionable insights. Advances in AI and machine learning are helping to address these challenges by improving data analysis and providing personalized recommendations based on individual health patterns.

Smartwatches and fitness trackers are poised to play an even greater role in digital healthcare by integrating advanced features such as continuous glucose monitoring, stress tracking, and early disease detection [75]. Innovations in sensor technology, battery life, and AI-driven analytics will further enhance their capabilities, making them indispensable tools for preventive health and chronic disease management. As these devices become more sophisticated and accessible, they have the potential to bridge the gap between traditional healthcare and everyday wellness, empowering individuals to take a proactive approach to their health while providing healthcare professionals with valuable data for remote monitoring and personalized care.

#### 2.1.3. Wearable Patches

Wearable patches represent a significant advancement in the field of bioelectronics, offering a flexible, adhesive, and minimally invasive solution for continuous health monitoring [76,77]. These patches are equipped with advanced sensors that can track vital signs such as electrocardiogram (ECG) signals, body temperature, and hydration levels, providing real-time data for both clinical and personal use [78,79,80]. Unlike bulkier wearable devices, wearable patches are designed to be lightweight, discreet, and comfortable for long-term wear, making them ideal for continuous monitoring in diverse settings, from hospitals to everyday life [81,82]. Their ability to conform to the skin and collect high-quality data without restricting movement has made them a promising tool for managing chronic conditions, post-operative care, and general wellness.

One of the key applications of wearable patches is in cardiovascular monitoring, where they provide continuous ECG tracking to detect arrhythmias, heart rate variability, and other cardiac abnormalities [82,83]. These patches often use flexible electrodes and advanced signal processing algorithms to ensure accurate and reliable data collection, even during physical activity. Additionally, wearable patches can monitor body temperature, which is crucial for detecting fevers, infections, or inflammatory conditions [84]. Some patches are also capable of measuring hydration levels through sweat analysis, providing insights into electrolyte balance and dehydration risks [85]. This multifunctional capability makes wearable patches particularly valuable for athletes, elderly patients, and individuals with chronic illnesses who require constant health oversight.

Despite their potential, wearable patches face challenges related to power supply, data accuracy, and user adherence. Many patches rely on small batteries or energy-harvesting technologies, which can limit their operational lifespan. Ensuring consistent data accuracy across different skin types, environmental conditions, and activity levels is another area of ongoing research [86,87]. Furthermore, user adherence can be influenced by factors such as skin irritation, patch durability, and ease of use. Advances in materials science, such as the development of biocompatible adhesives and stretchable electronics, are helping to address these challenges, making patches more comfortable and reliable for long-term wear.

**Figure 3:**
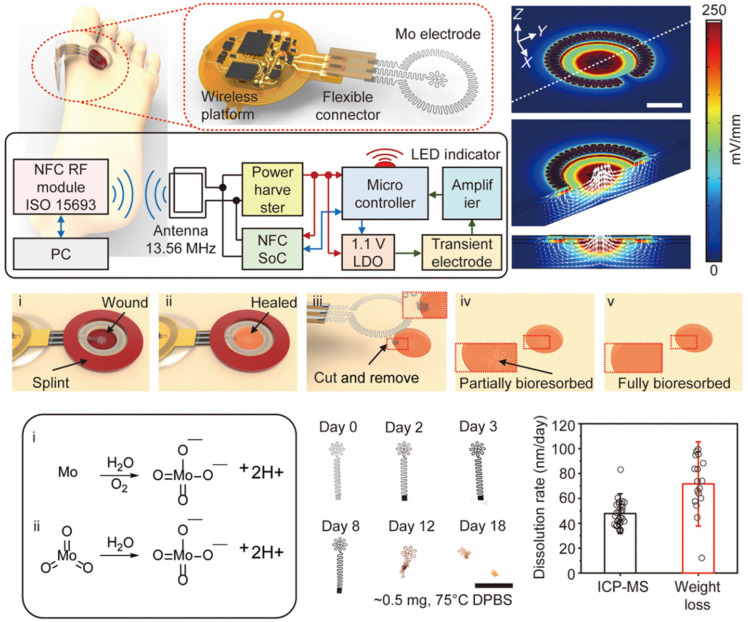
Materials and structural design of a fully bioresorbable, wireless, and battery-free electrotherapy system. For i–v, photographs capturing the dissolution of an inner electrode structure with interconnects at various immersion times in Dulbecco’s phosphate-buffered saline (DPBS; pH 7.4) at 75 °C. Scale bar: 5 mm. Reproduced with permission [92]. Copyright 2023, Creative Commons Attribution License 4.0 (CC BY).

The future of wearable patches is bright, with ongoing innovations aimed at expanding their capabilities and applications. Researchers are exploring the integration of additional sensors to monitor biomarkers like glucose, lactate, and cortisol, further enhancing their utility in personalized healthcare [88,89], as exemplified in Figure 3. The incorporation of wireless connectivity and AI-driven analytics will enable real-time data transmission and actionable insights, bridging the gap between patients and healthcare providers. As these technologies mature, wearable patches have the potential to revolutionize remote patient monitoring, reduce healthcare costs, and improve outcomes by enabling early detection and intervention [90,91]. By combining convenience, functionality, and precision, wearable patches are set to become a cornerstone of next-generation digital healthcare solutions.

#### 2.1.4. Implantable Devices

**Figure 4:**
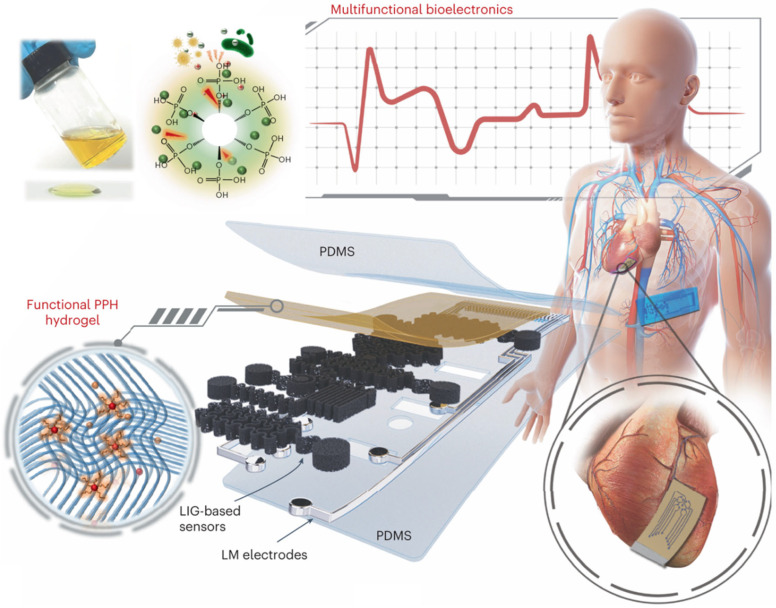
Design of stretchable graphene–hydrogel nanocomposites. Illustration of the structure of thin, stretchable nanocomposites enhanced with antibacterial and biocompatible PPH hydrogel, suitable for wearable and implantable bioelectronics. Reproduced with permission [103]. Copyright 2024, Springer Nature Limited.

Implantable devices represent the cutting edge of bioelectronics, offering long-term monitoring and therapeutic capabilities for a wide range of medical conditions (Figure 4) [79]. These advanced devices, such as pacemakers, neural stimulators, and continuous glucose monitors (CGMs), are designed to be embedded within the body, providing precise and continuous data or delivering targeted treatments [93,94]. Unlike external wearable devices, implantable bioelectronics operate seamlessly within the body, minimizing user intervention and offering a higher degree of accuracy and reliability [94,95]. They are particularly valuable for managing chronic conditions, such as cardiovascular diseases, diabetes, and neurological disorders, where continuous monitoring and timely interventions are critical.

The most well-known example of implantable devices is the pacemaker, which regulates abnormal heart rhythms by delivering electrical impulses to the heart muscle [96]. Similarly, neural stimulators, such as deep brain stimulators (DBS) and vagus nerve stimulators (VNS), are used to treat conditions like Parkinson’s disease, epilepsy, and chronic pain by modulating neural activity [97]. CGMs are another breakthrough, providing real-time glucose level readings for individuals with diabetes, reducing the need for frequent finger-prick tests [98,99]. These devices not only improve quality of life but also enable personalized and data-driven healthcare, allowing clinicians to tailor treatments based on real-time physiological data.

However, implantable devices still face challenges related to biocompatibility, power supply, and long-term reliability. Ensuring that these devices are safe and compatible with the body’s tissues is critical to prevent adverse reactions such as inflammation or rejection [100]. Power supply is another significant concern, as many implantable devices rely on batteries with limited lifespans, necessitating surgical replacement. Researchers are exploring innovative solutions such as energy harvesting from body heat or motion, as well as wireless charging technologies, to address this issue. Additionally, the long-term durability and functionality of these devices must be ensured to avoid malfunctions or the need for frequent replacements [101].

Advances in materials science, miniaturization, and wireless communication are unlocking the remarkable potential of implantable devices [102]. Emerging technologies, such as bioresorbable electronics that dissolve after fulfilling their purpose, are opening new possibilities for temporary monitoring and treatment. The integration of AI and machine learning will further enhance the capabilities of these devices, enabling predictive analytics and adaptive therapies. As implantable devices become more sophisticated and accessible, they will play an increasingly vital role in personalized medicine, offering hope for improved outcomes and a better quality of life for patients with chronic and complex medical conditions. By bridging the gap between technology and biology, implantable bioelectronics are set to redefine the future of healthcare.

#### 2.1.5. Smart Textiles

**Figure 5:**
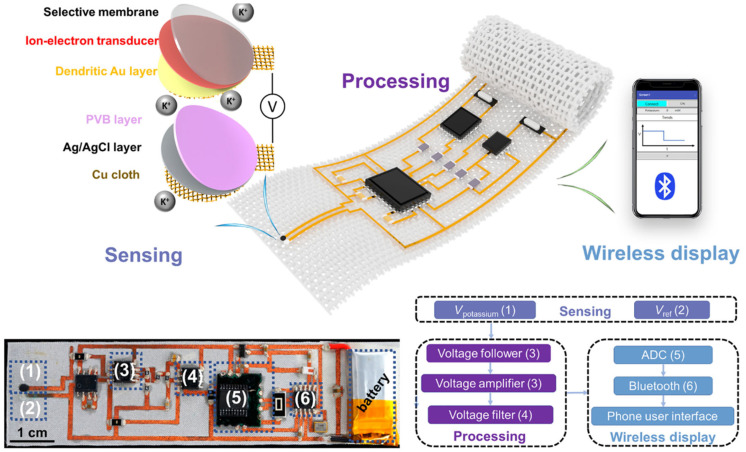
Image and schematic illustration of the monolithically integrated in-textile wristband. Schematic of the textile wristband designed for wireless sweat K^+^ analysis. Photograph and schematic of the flexible patch (2.5 cm × 9 cm). Logical flow of the system design. Reproduced with permission [107]. Copyright 2023, Creative Commons Attribution License 4.0 (CC BY).

Smart clothing represents a revolutionary fusion of textiles and technology, embedding biosensors directly into fabrics to enable seamless and unobtrusive health monitoring [104]. Unlike traditional wearable devices, smart clothing integrates sensors into everyday garments such as shirts, bras, socks, and even underwear, allowing users to monitor their health without the need for additional accessories (Figure 5). These garments are designed to measure a variety of biomarkers, including cortisol levels, electrolytes, heart rate, and muscle activity, providing continuous and real-time data [105]. By blending functionality with comfort, smart clothing offers a discreet and user-friendly approach to health monitoring, making it ideal for both everyday wellness tracking and medical applications [106].

Smart clothing allows monitoring of physiological signals in a natural and non-invasive manner. For example, shirts with embedded biosensors can measure heart rate and respiratory patterns during physical activity, while bras equipped with sensors can track stress levels by monitoring cortisol in sweat [108]. Similarly, socks with pressure sensors can analyze gait and detect early signs of mobility issues or diabetic foot ulcers. The integration of conductive fibers and flexible electronics into fabrics ensures that these garments remain comfortable and durable, even after repeated use and washing. This seamless integration of technology into clothing eliminates the need for bulky devices, making health monitoring more accessible and convenient.

Although smart clothing holds great promise, it still encounters challenges in sensor accuracy, reliable power supply, and efficient data processing [109]. Ensuring that biosensors embedded in fabrics provide reliable and consistent measurements across different body types, activity levels, and environmental conditions is a significant hurdle. Powering these sensors without compromising the comfort or flexibility of clothing is another challenge, with researchers exploring solutions such as energy-harvesting textiles and flexible batteries. Additionally, the vast amount of data generated by smart clothing requires efficient processing and analysis, often leveraging AI and cloud computing to provide actionable insights. Addressing these challenges will be critical to the widespread adoption and success of smart clothing in healthcare [110].

Smart clothing holds great promise for the future, driven by continuous innovations in materials science, sensor integration, and data analytics. Researchers are exploring the integration of additional functionalities, such as temperature regulation, UV protection, and even drug delivery, into smart fabrics [111]. The development of washable and stretchable electronics will further enhance the durability and usability of these garments. As smart clothing becomes more sophisticated and affordable, it has the potential to transform healthcare by enabling continuous, real-time monitoring in a way that is both practical and unobtrusive. By merging fashion with technology, smart clothing is poised to become a cornerstone of personalized and preventive healthcare, empowering individuals to take control of their health in a seamless and integrated manner.

### 2.2. Materials Properties

The performance, comfort, and durability of wearable bioelectronics are heavily influenced by the materials and fabrication techniques used in their design [112,113]. Recent advancements in materials science and engineering have enabled the development of devices that are not only highly functional but also comfortable and reliable for long-term use, see Table 2. Key innovations in this area include the use of flexible and stretchable materials, biocompatible polymers, durable encapsulation methods, and miniaturization technologies [114,115]. These advancements have paved the way for wearable bioelectronics that seamlessly integrate with the human body, providing continuous health monitoring without compromising user comfort or device performance.

Flexibility and stretchability are critical properties for wearable bioelectronics, as they allow devices to conform to the skin or other body surfaces without causing discomfort or restricting movement [116,117]. Materials such as polymers, elastomers, and nanomaterials (e.g., graphene and carbon nanotubes) have been instrumental in achieving these properties. For instance, elastomers like polydimethylsiloxane (PDMS) and polyurethane offer excellent elasticity and mechanical resilience, making them ideal for wearable patches and smart textiles [118]. Nanomaterials, such as graphene and carbon nanotubes, provide exceptional electrical conductivity and mechanical strength while maintaining flexibility [119]. These materials enable the development of sensors and circuits that can stretch, bend, and twist with the body, ensuring accurate data collection even during physical activity.

Biocompatibility is another essential consideration for wearable and implantable bioelectronics, as these devices are in direct contact with the skin or internal tissues [120]. Materials used in these devices must be non-toxic, non-irritating, and resistant to causing allergic reactions or inflammation. Biocompatible polymers like PDMS and hydrogels are widely used due to their softness, flexibility, and compatibility with biological tissues. Hydrogels, in particular, are highly water-absorbent and can mimic the mechanical properties of human tissue, making them suitable for applications such as wound healing and drug delivery. Additionally, surface modifications and coatings are often applied to enhance biocompatibility and prevent adverse reactions, ensuring that devices can be worn or implanted safely for extended periods [121].

Durability and miniaturization are also critical factors in the design of wearable bioelectronics. Devices must withstand mechanical stress, moisture, temperature variations, and repeated use without degrading in performance. Advanced encapsulation techniques, such as thin-film coatings and multilayer barriers, protect sensitive electronic components from environmental factors like sweat, humidity, and mechanical strain [24,33]. At the same time, advances in microfabrication and nanotechnology have enabled the development of compact, lightweight devices that are comfortable for continuous wear. Techniques such as photolithography, 3D printing, and laser patterning allow for the precise fabrication of miniaturized sensors and circuits, reducing the overall size and weight of wearable devices. These innovations ensure that wearable bioelectronics are not only durable and reliable but also unobtrusive and user-friendly, making them ideal for everyday health monitoring and long-term medical applications [122].

### 2.3. Power Sources and Energy Harvesting Methods

Sustained operation of wearable bioelectronics relies heavily on efficient power management solutions, as traditional batteries are often bulky, rigid, and have limited lifespans, making them unsuitable for long-term or continuous use [121,123]. To address these limitations, researchers and engineers have developed innovative power sources and energy harvesting methods that enable wearable devices to operate efficiently without frequent recharging or replacement. These advancements are critical for ensuring that wearable bioelectronics remain lightweight, comfortable, and functional for extended periods, particularly in applications such as health monitoring and medical diagnostics [124].

**Figure 6:**
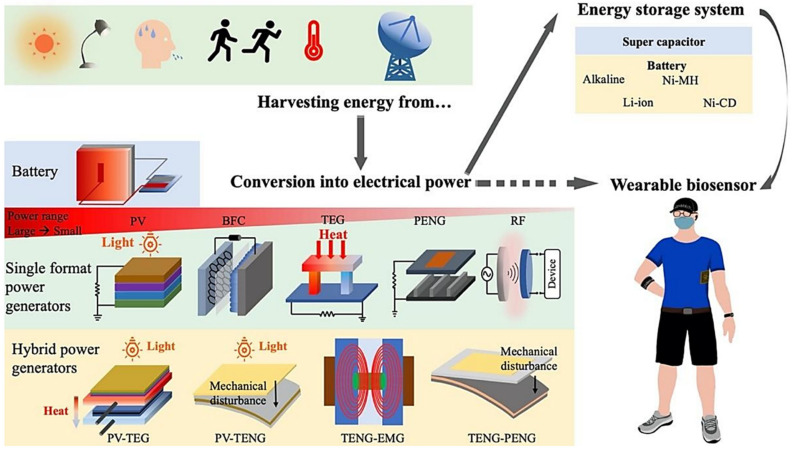
Schematic flow chart of self-powering smart wearable sensors. The process involves harvesting energy from various sources, converting it into electrical power, storing the energy, and using it to power wearable sensors. Energy Sources: PV (Photovoltaic); BFCs (Biofuel Cells); TEGs (Thermoelectric Generators); PENGs (Piezoelectric Nanogenerators); TENGs (Triboelectric Nanogenerators); RF (Radio Frequency); EMGs (Electromagnetic Generators). Reproduced with permission [131]. Copyright 2021, Creative Commons Attribution License 4.0 (CC BY).

Energy harvesting techniques have emerged as a promising solution to power wearable bioelectronics by converting ambient energy from the environment or the user’s body into electrical energy [114,125] (see Figure 6). Piezoelectric materials, for example, generate electricity from mechanical energy, such as body movements or vibrations, making them ideal for devices worn during physical activity. Thermoelectric materials leverage temperature differences, such as between the body and the surrounding environment, to produce power. Photovoltaic cells, on the other hand, harvest solar energy, providing a renewable power source for outdoor wearables [126]. These energy harvesting methods not only reduce reliance on traditional batteries but also enable self-sustaining devices that can operate indefinitely under the right conditions.

Flexible batteries represent another significant advancement in powering wearable bioelectronics [127]. Unlike conventional rigid batteries, flexible batteries are made from advanced materials such as thin-film lithium ion or solid-state electrolytes, allowing them to bend and stretch without losing functionality. These batteries can be seamlessly integrated into wearable fabrics or patches, maintaining the device’s comfort and flexibility. Additionally, researchers are exploring biodegradable and eco-friendly battery materials to address environmental concerns [128]. Wireless charging technologies, such as inductive and resonant charging, further enhance the usability of wearable devices by eliminating the need for physical connectors. These methods enable convenient and efficient recharging, often through portable charging pads or even clothing embedded with charging coils.

To complement these power sources, the integration of low-power electronics is essential for minimizing energy consumption and extending battery life [129,130]. Advances in low-power sensors, microprocessors, and communication modules have significantly reduced the energy requirements of wearable bioelectronics. For instance, ultra-low-power Bluetooth and near-field communication (NFC) technologies enable efficient data transmission with minimal energy expenditure. Similarly, energy-efficient algorithms and sleep modes optimize device operation, ensuring that power is used only when necessary. By combining energy harvesting, flexible batteries, wireless charging, and low-power electronics, wearable bioelectronics can achieve a balance between functionality, comfort, and sustainability, paving the way for their widespread adoption in digital healthcare and beyond.

### 2.4. Signal Processing and Data Acquisition

Wearable bioelectronics rely on advanced signal processing and data acquisition techniques to ensure accurate, reliable, and meaningful health data collection. These devices often operate in dynamic and noisy environments, where external interference, motion artifacts, and signal degradation can compromise data quality. To address these challenges, sophisticated signal processing methods are employed to filter out noise, amplify weak signals, and extract relevant information. For example, filtering techniques such as low-pass, high-pass, and band-pass filters are used to remove unwanted frequencies, while adaptive algorithms can dynamically adjust to changing conditions [132,133]. Signal amplification ensures that weak physiological signals, such as those from ECG or photoplethysmography (PPG), are captured with sufficient clarity for analysis. These techniques are critical for maintaining the accuracy and reliability of wearable devices, particularly in applications like cardiac monitoring or stress detection [134,135].

Multimodal sensing is another key aspect of wearable bioelectronics, enabling the integration of multiple sensor types to provide a comprehensive view of an individual’s health [136]. By combining data from optical, electrochemical, and mechanical sensors, these devices can monitor a wide range of parameters, such as heart rate, blood oxygen levels, glucose concentration, and physical activity. For instance, a smartwatch might use optical sensors for PPG-based heart rate monitoring, accelerometers for tracking movement, and temperature sensors for detecting fever or inflammation. Multimodal sensing not only enhances the richness of the data but also improves accuracy by cross-validating measurements from different sources [12]. This approach is particularly valuable for complex health monitoring tasks, such as detecting early signs of chronic diseases or assessing overall wellness.

Real-time data transmission is essential for enabling timely analysis and decision-making in wearable bioelectronics [1,24,137]. Wireless communication protocols, such as Bluetooth Low Energy (BLE), Wi-Fi, and near-field communication (NFC), facilitate the seamless transfer of data from wearable devices to external platforms, such as smartphones, tablets, or cloud-based systems. BLE, in particular, is widely used due to its low power consumption and ability to transmit data over short distances efficiently. Once transmitted, the data can be processed using advanced algorithms, including machine learning and artificial intelligence, to generate actionable insights. Real-time data transmission also enables remote monitoring by healthcare providers, allowing for timely interventions and personalized care. By combining robust signal processing, multimodal sensing, and efficient data transmission, wearable bioelectronics can deliver accurate, real-time health monitoring, paving the way for more proactive and personalized healthcare solutions.

However, the implementation of BLE/NFC protocols in wearable bioelectronics introduces critical security vulnerabilities that demand careful consideration: (1) eavesdropping risks in public communication channels, (2) data integrity threats during transmission, and (3) authentication challenges in multi-device ecosystems. These technical limitations directly inform the ethical and regulatory frameworks discussed in Section 5.1 (HIPAA/GDPR compliance requirements) and motivate the encryption solutions presented in Section 5.1, including emerging privacy-preserving algorithms for wearable AI systems. Recent studies demonstrate improved BLE security frameworks, while FDA cybersecurity guidance (2023) provides critical benchmarks for medical-grade data protection, collectively addressing these vulnerabilities through standardized best practices.

## 3. Artificial Intelligence

**Figure 7:**
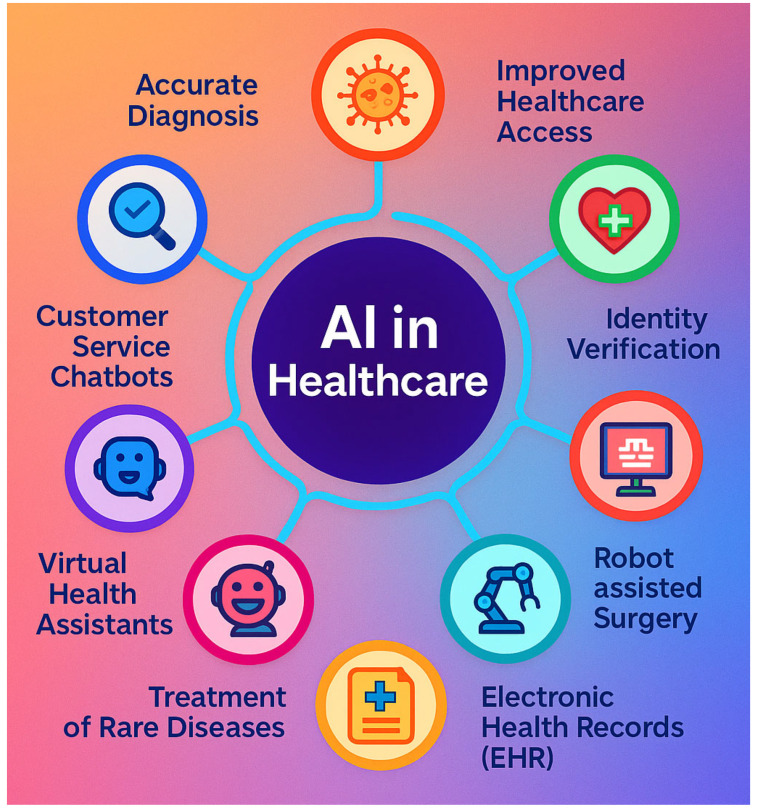
The broad applications of AI in healthcare, including disease treatment, accurate diagnosis, electronics health records, etc.

AI has become a cornerstone of modern wearable bioelectronics, enabling the transformation of raw sensor data into actionable insights by leveraging advanced algorithms such as machine learning (ML) and deep learning (DL) [138]. These algorithms allow wearable devices to analyze complex datasets, identify patterns, and make predictions, enhancing their ability to monitor and manage health in real time. For instance, supervised learning models can classify ECG signals to detect arrhythmias, while unsupervised learning techniques can cluster data to identify trends in patient health. Deep learning, with its ability to process high-dimensional data, is particularly effective in tasks like analyzing multi-sensor inputs or interpreting medical images. Additionally, AI-driven data processing techniques, such as noise reduction, feature extraction, and anomaly detection, ensure that the data collected is accurate and meaningful [139]. The integration of AI with edge computing further amplifies these capabilities by enabling real-time analytics directly on the device, reducing latency, enhancing privacy, and minimizing energy consumption. This combination of AI and edge computing allows wearable bioelectronics to provide immediate feedback, such as alerting users to abnormal heart rhythms or predicting hypoglycemic episodes, making them indispensable tools for personalized and proactive healthcare [140]. As AI technologies continue to evolve, they will further enhance the intelligence, efficiency, and adaptability of wearable devices, driving the future of digital healthcare, as demonstrated by Figure 7.

### 3.1. Overview of AI Algorithms in Healthcare

AI algorithms, particularly ML and DL, are revolutionizing healthcare by enabling the analysis of complex datasets and the extraction of meaningful patterns, as summarized in Table 3. These algorithms empower wearable bioelectronics to perform advanced tasks such as anomaly detection, predictive analytics, and personalized recommendations, transforming raw sensor data into actionable health insights. By leveraging large datasets and sophisticated computational techniques, AI enhances the accuracy, adaptability, and intelligence of wearable devices, making them indispensable tools for modern healthcare.

Supervised Learning is one of the most widely used AI techniques in healthcare, particularly for classification and regression tasks [141,142]. In classification, supervised learning algorithms are trained on labeled datasets to categorize data into predefined classes [143]. For example, ECG data can be classified to detect arrhythmias or atrial fibrillation, while accelerometer data can be used to identify specific physical activities like walking, running, or sleeping, with a sensitivity of 96%. Regression tasks, on the other hand, involve predicting continuous outcomes, such as estimating blood glucose levels or forecasting the risk of cardiovascular events. Supervised learning models, such as support vector machines (SVMs), random forests, and logistic regression, are highly effective in these applications due to their ability to learn from labeled data and make accurate predictions. For example, random forests are particularly effective for real-time fall detection in wearable devices due to their ability to process multi-sensor inertial data (accelerometer, gyroscope) with low computational overheads. Studies demonstrate >90% detection accuracy within 100 ms on ultra-low-power microcontrollers (e.g., ARM Cortex-M4), making them ideal for edge deployment in elderly monitoring systems. Their inherent feature, importance analysis, also helps optimize sensor configurations, reducing power consumption by up to 40% compared to deep learning alternatives

Unsupervised learning plays a critical role in identifying hidden patterns or structures in data without the need for labeled examples [144,145]. This approach is particularly useful for clustering and dimensionality reduction, enabling the discovery of natural groupings or trends in health data. For instance, unsupervised learning algorithms like k-means clustering or hierarchical clustering can group patients based on similar health profiles, such as risk factors for diabetes or responses to a specific treatment, achieving sensitivity up to 98% and specificity up to 96%. Dimensionality reduction techniques, such as principal component analysis (PCA) or t-distributed stochastic neighbor embedding (t-SNE), help simplify complex datasets by extracting the most relevant features, making it easier to visualize and interpret health data [146]. These capabilities are invaluable for personalized medicine, where understanding individual variations is key to effective treatment.

Deep Learning, a subset of machine learning, has gained significant attention for its ability to process large-scale, high-dimensional data using neural networks. Deep learning models, such as convolutional neural networks (CNNs) and recurrent neural networks (RNNs), excel at tasks like analyzing medical images, interpreting multi-sensor data streams, and processing natural language. For example, 1D CNNs have become the standard for analyzing raw ECG/PPG signals in smartwatches and patches. Optimized architectures like TinyCNN achieve 95–98% arrhythmia detection sensitivity while operating under 5 mW power budgets—critical for continuous monitoring. Recent FDA-cleared devices (e.g., AliveCor KardiaMobile) utilize these models with spectral attention mechanisms to maintain performance during motion artifacts, demonstrating the clinical viability of this approach. Also, CNNs can analyze retinal images to detect diabetic retinopathy, while RNNs can interpret sequential data, such as CGM readings, to predict future trends (sensitivity: 78–95%; specificity: 85–95) [147]. The strength of deep learning lies in its ability to automatically learn hierarchical features from raw data, eliminating the need for manual feature engineering and enabling more accurate and robust predictions.

Reinforcement learning (RL) is another powerful AI technique that enables adaptive decision-making in dynamic environments [148,149]. In healthcare, RL is used to optimize treatment strategies and personalize interventions based on real-time feedback. For example, RL algorithms can optimize insulin dosages in closed-loop systems for diabetes management or adjust stimulation parameters in neural implants for Parkinson’s disease. Meanwhile, variational autoencoders address the scarcity of labeled data in continuous glucose monitoring by learning normal physiological patterns from unlabeled datasets. Their unsupervised framework detects hypoglycemic events with 85–90% precision while adapting to individual metabolic variations through few-shot learning. When deployed on wearable processors, quantized versions (<200 KB) reduce false alarms by 30% compared to threshold-based systems, showcasing their potential for personalized diabetes management. These systems learn by interacting with the environment and receiving rewards or penalties based on their actions, allowing them to continuously improve their performance. Reinforcement learning is particularly promising for applications requiring real-time adaptation, such as personalized therapy and chronic disease management, where individualized treatment plans are essential for optimal outcomes, with sensitivity up to 97% and selectivity up to 93%.

By leveraging these AI algorithms, wearable bioelectronics can provide accurate, personalized, and real-time health insights, empowering users to take proactive control of their health [150]. As AI technologies continue to evolve, they will further enhance the capabilities of wearable devices, enabling more sophisticated applications and driving the future of digital healthcare.

### 3.2. Data Processing and Analysis Techniques

Wearable bioelectronics generate vast amounts of data from various sensors, such as heart rate monitors, accelerometers, and glucose sensors. To transform this raw data into actionable insights, advanced data processing and analysis techniques are essential. AI plays a critical role in this process, enabling wearable devices to filter noise, extract meaningful features, recognize patterns, and predict future health events [151,152]. These capabilities allow wearable bioelectronics to go beyond simple data collection, providing users and healthcare providers with real-time, actionable insights for better decision-making.

Noise reduction and signal enhancement are crucial for ensuring the accuracy and reliability of data collected by wearable devices [153,154]. In dynamic environments, sensor data can be contaminated by noise and artifacts, such as motion artifacts in ECG signals or interference in optical heart rate measurements. AI algorithms, such as adaptive filters and wavelet transforms, are used to isolate and remove these unwanted components, enhancing the quality of the signal. For example, deep learning models can be trained to distinguish between true physiological signals and noise, ensuring that the data used for analysis is clean and accurate. This step is particularly important for applications like cardiac monitoring, where even small errors can lead to incorrect diagnoses or missed alerts [155].

Feature extraction is the process of identifying and isolating relevant information from raw sensor data [156]. AI algorithms are used to extract features that are meaningful for health monitoring, such as heart rate variability (HRV) from ECG signals, respiratory rate from chest movements, or sleep stages from accelerometer data. Techniques like Fourier transforms, principal component analysis (PCA), and time–frequency analysis are commonly used for this purpose. For instance, HRV features can provide insights into autonomic nervous system activity, while sleep stage classification can help diagnose sleep disorders. By focusing on these key features, AI reduces the complexity of the data, making it easier to analyze and interpret [157].

Pattern recognition is another critical capability enabled by AI, allowing wearable devices to detect anomalies, trends, and early signs of disease. Machine learning models, such as support vector machines (SVMs) and neural networks, are trained to recognize patterns in the data that may indicate health issues. For example, AI can identify irregular heart rhythms (arrhythmias) from ECG data or detect early signs of neurodegenerative diseases like Parkinson’s from movement patterns [158]. These models can also track trends over time, such as changes in blood pressure or glucose levels, providing early warnings for potential health risks. By detecting anomalies and trends in real time, AI enables timely interventions, improving outcomes for patients.

Predictive analytics takes data analysis a step further by forecasting future health events based on historical and real-time data [159]. AI models, such as recurrent neural networks (RNNs) and long short-term memory (LSTM) networks, are particularly effective for time-series data, enabling predictions of hypoglycemic episodes in diabetic patients or the onset of cardiovascular events. For example, CGMs can use predictive analytics to alert users of impending low blood sugar levels, allowing them to take preventive action. Similarly, wearable devices can predict the risk of heart attacks by analyzing trends in heart rate, blood pressure, and activity levels [160]. These predictive capabilities empower users to take proactive control of their health, reducing the likelihood of adverse events and improving overall well-being.

By leveraging these data processing and analysis techniques, AI transforms wearable bioelectronics into intelligent systems capable of providing real-time, actionable insights. These advancements not only enhance the accuracy and reliability of health monitoring but also enable personalized and proactive healthcare, empowering users and healthcare providers to make better-informed decisions [161]. As AI technologies continue to evolve, they will further enhance the capabilities of wearable devices, driving the future of digital healthcare.

### 3.3. Integration of AI with Edge Computing

The integration of AI with edge computing represents a transformative advancement for wearable bioelectronics, enabling real-time data analysis directly on the device rather than relying on external servers or cloud platforms [162,163]. This approach addresses critical challenges such as latency, privacy, energy efficiency, and scalability, making wearable devices more responsive, secure, and user-friendly. By processing data locally, edge AI empowers wearable bioelectronics to deliver immediate insights and actionable feedback, enhancing their utility in both personal health monitoring and clinical applications.

Edge computing enables real-time health monitoring by processing data locally on wearable devices, reducing latency (<100 ms) for critical applications like fall detection or arrhythmia alerts while enhancing privacy through minimized cloud transmission of sensitive physiological data. This approach also improves power efficiency—enabling weeks-long operation between charges—by eliminating energy-intensive data streaming and allowing offline functionality in low-connectivity settings. Practical implementations (e.g., on-device ECG analysis in smartwatches) demonstrate 30–50% reductions in both cloud dependency and false alarms compared to cloud-dependent systems.

Real-time analytics is one of the most significant benefits of integrating AI with edge computing [164]. Wearable devices equipped with edge AI capabilities can process sensor data instantly, providing users with immediate feedback and alerts. For example, a wearable ECG monitor can analyze heart rhythm data in real time to detect arrhythmias and notify the user or healthcare provider without delay. Similarly, devices like smartwatches can use edge AI to monitor physical activity and provide instant feedback on exercise intensity or recovery needs [165]. This real-time processing is particularly critical for applications requiring immediate intervention, such as detecting falls in elderly users or monitoring vital signs in critical care settings. By eliminating the need to transmit data to external servers, edge AI ensures that responses are timely and actionable.

Privacy and security are paramount in healthcare, and edge computing significantly enhances data protection by minimizing the need for data transmission [166,167]. When sensitive health data is processed locally on the device, the risk of exposure during transmission or storage on external servers is greatly reduced. This is especially important for wearable bioelectronics, which often collect highly personal information such as heart rate, glucose levels, or sleep patterns. Edge AI ensures that data remains on the device, with only anonymized or aggregated results being shared with healthcare providers or cloud platforms when necessary. This localized approach not only safeguards user privacy but also builds trust in wearable technologies, encouraging wider adoption.

Energy efficiency is another critical advantage of edge AI, as it reduces the power consumption of wearable devices [168,169]. Transmitting large volumes of data to external servers for processing can drain battery life quickly, limiting the usability of wearable bioelectronics. By performing data analysis locally, edge AI minimizes the need for constant data transmission, significantly extending battery life. Additionally, optimized AI models and hardware accelerators, such as tensor processing units (TPUs) or neuromorphic chips, are designed to perform complex computations with minimal energy consumption. These advancements ensure that wearable devices remain lightweight, compact, and functional for extended periods, enhancing user convenience and compliance.

Scalability is a key consideration for the widespread adoption of wearable bioelectronics, and edge computing enables devices to operate independently without overloading cloud infrastructure. By processing data locally, wearable devices reduce the demand for bandwidth and computational resources on external servers, making it easier to deploy these technologies at scale. For example, edge AI can enable real-time stress detection using heart rate variability analysis or fall detection using accelerometer data, even in remote or resource-constrained settings [170]. This scalability is particularly important for applications in global health, where access to reliable internet connectivity or cloud services may be limited. By leveraging edge AI, wearable bioelectronics can deliver consistent and reliable performance across diverse populations and environments [171].

**Figure 8:**
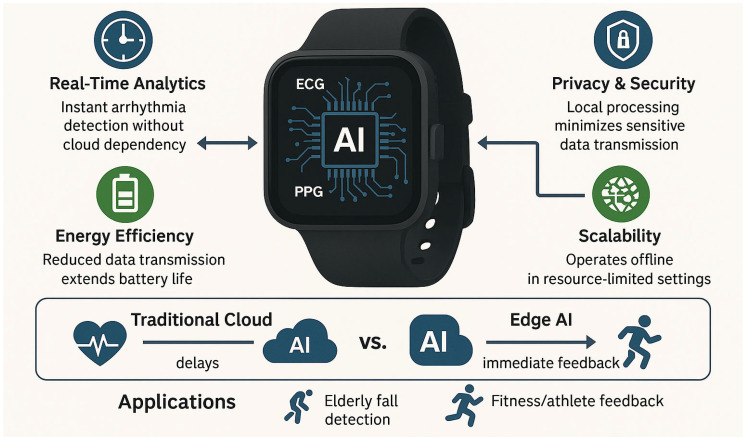
Integration of AI with edge computing for wearable bioelectronics devices.

In summary, the integration of AI with edge computing is a game-changer for wearable bioelectronics, enabling real-time analytics, enhancing privacy and security, improving energy efficiency, and ensuring scalability (Figure 8). These advancements make wearable devices more intelligent, responsive, and user-friendly, paving the way for their widespread adoption in digital healthcare. As edge AI technologies continue to evolve, they will further enhance the capabilities of wearable bioelectronics, driving innovation and improving health outcomes for individuals worldwide.

### 3.4. AI–Biosensor Fusion Framework: Methodological Pillars

#### 3.4.1. AI-Enhanced Sensing Principles

The first pillar establishes AI-driven techniques to fundamentally augment biosensing capabilities (Table 4). Deep convolutional neural networks (1D-CNNs) now achieve >90% noise suppression in raw biosignals while preserving critical physiological features through learned wavelet transforms. For multi-modal systems, hybrid transformer architectures with cross-attention mechanisms demonstrate 40–60% specificity improvements by intelligently fusing electrochemical, optical, and mechanical sensor streams. These approaches are complemented by adaptive sampling controllers, where reinforcement learning agents dynamically adjust measurement parameters (e.g., 0.1–100 Hz sampling rates) based on real-time biomarker kinetics, reducing power consumption by 75% without compromising data fidelity. The framework provides standardized preprocessing pipelines and quality metrics (SNR gain, feature preservation index) to benchmark these enhancements across different biosensor classes.

#### 3.4.2. Closed-Loop Optimization Methods

This pillar introduces AI as a co-design partner throughout the biosensor lifecycle. Graph neural networks accelerate transducer material discovery by predicting nanostructure–property relationships, experimentally validated to identify 15 novel sensing composites with 10× faster development cycles. At the interface level, generative adversarial networks (GANs) propose 3D bioreceptor configurations demonstrating 35% higher binding affinity than human-designed counterparts through evolutionary optimization. System-level co-optimization is achieved via neural architecture searches (NASs) that simultaneously balance response time (<50 ms target), sensitivity (pM-level detection), and energy efficiency (<1 mJ/measurement)—a multi-objective optimization problem previously intractable with conventional methods. The framework includes validation protocols to ensure AI-proposed designs meet clinical reliability standards (e.g., <5% CV across 1000 test cycles).

#### 3.4.3. Intelligent Diagnostic Paradigms

Moving beyond sensing hardware, this pillar redefines diagnostic workflows through embedded AI. Context-aware analysis engines incorporate federated learning to personalize biomarker interpretation using wearer-specific baselines while maintaining privacy through distributed model updates. Predictive maintenance modules employ LSTM networks to forecast sensor degradation (mean absolute error <0.5%), triggering self-calibration routines that extend operational stability by 3–5×. The framework also establishes hybrid edge-cloud architectures where ultra-lightweight models (<100 kB) handle time-critical detection locally, while resource-intensive analyses (e.g., longitudinal trend prediction) occur in secure cloud modules with medical-grade verification. Diagnostic reliability is ensured through uncertainty quantification layers that flag low-confidence predictions for clinician review.

#### 3.4.4. Implementation Roadmap

The final pillar translates these concepts into practice through a staged deployment pathway. Phase 1 focuses on modular AI toolkits (Python/PyTorch) version 3.13.5 for benchtop biosensor development, featuring pretrained models for common noise profiles and interference patterns. Phase 2 addresses embedded implementation via TensorFlow Lite modules optimized for wearable processors (ARM Cortex-M7/RISC-V), including memory-efficient quantization techniques. Phase 3 establishes clinical validation protocols, with FDA-aligned documentation templates for AI model governance (data drift monitoring, failure mode analysis). The complete framework is supported by an open registry of benchmark datasets and performance standards (e.g., minimum 80% specificity at 99% sensitivity for critical biomarkers), creating a shared development ecosystem for the research community.

## 4. Applications in Digital Healthcare

Wearable bioelectronics, powered by AI and advanced sensor technologies, are transforming digital healthcare by enabling a wide range of applications. These devices provide continuous, real-time monitoring of health parameters, facilitate early detection of diseases, and offer personalized insights for better health management (see Table 5). This section explores the diverse applications of wearable bioelectronics in digital healthcare, focusing on health monitoring, diagnosis, and prognosis.

### 4.1. Health Monitoring

**Figure 9:**
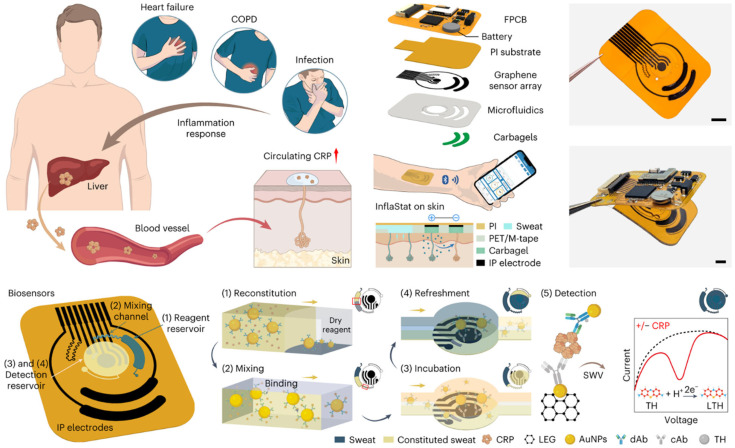
Non-Invasive, Wireless Wearable Biosensor for Continuous Electrochemical Monitoring of Inflammation. Reproduced with permission [175]. Copyright 2023, Springer Nature Limited.

Health monitoring is one of the most prominent and transformative applications of wearable bioelectronics, enabling individuals to track their vital signs and overall well-being in real time, as shown in Figure 9. These devices provide continuous, non-invasive monitoring of key health metrics, empowering users to take proactive steps toward maintaining their health and preventing potential issues. By leveraging advanced sensors, AI algorithms, and seamless connectivity, wearable bioelectronics are revolutionizing the way we monitor and manage health. This section delves deeper into the key areas of health monitoring, including continuous monitoring of vital signs, early detection of anomalies, and mental health monitoring, highlighting their significance and impact on digital healthcare.

#### 4.1.1. Continuous Monitoring of Vital Signs

Wearable bioelectronics are revolutionizing health monitoring by providing continuous, real-time tracking of vital signs such as heart rate, blood pressure, glucose levels, SpO2, and body temperature. These devices leverage advanced sensors to deliver accurate and non-invasive measurements, empowering users to stay informed about their physiological state [172]. For instance, optical sensors in smartwatches use PPG to measure heart rate by detecting changes in blood flow, offering insights into cardiovascular health and exercise intensity. Similarly, wearable patches and cuffs employ piezoelectric or oscillometric sensors to monitor blood pressure continuously, helping users manage hypertension and reduce the risk of complications like stroke or heart attack. CGMs use electrochemical sensors to track glucose levels in interstitial fluid, enabling diabetic patients to manage their condition effectively. Additionally, optical sensors measure SpO2 levels to assess respiratory health, while wearable thermometers provide continuous body temperature monitoring for early detection of fevers or infections.

The ability to monitor these vital signs continuously allows for the early detection of abnormalities, such as irregular heart rhythms, hypoglycemia, or respiratory issues. By providing timely alerts and actionable insights, wearable bioelectronics enable users to take proactive steps, such as seeking medical attention or adjusting their lifestyle, before minor issues escalate into serious health problems [173,174]. This real-time monitoring not only enhances individual health management but also reduces the burden on healthcare systems by preventing avoidable complications. As wearable technologies continue to advance, they are becoming indispensable tools for maintaining health and well-being, offering personalized and preventive care that empowers users to take control of their health.

#### 4.1.2. Detection of Anomalies and Early Warning Systems for Chronic Diseases

Wearable devices equipped with AI algorithms are transforming the management of chronic diseases by detecting anomalies in health data and providing early warnings. These systems analyze patterns in data to identify deviations from normal ranges, enabling timely interventions that can prevent complications and improve outcomes [176]. For example, ECG monitors in wearables can detect irregular heart rhythms (arrhythmias) such as atrial fibrillation, which, if left untreated, can lead to stroke or heart failure. AI algorithms analyze ECG data in real time, alerting users to seek medical attention when abnormalities are detected. Similarly, CGMs use predictive analytics to forecast hypoglycemic or hyperglycemic episodes, allowing diabetic patients to take preventive measures, such as adjusting insulin doses or consuming carbohydrates, to avoid dangerous fluctuations [177].

In addition to cardiovascular and diabetes management, wearable bioelectronics play a crucial role in monitoring respiratory health. Devices that measure respiratory rate can detect early signs of conditions like chronic obstructive pulmonary disease (COPD) or sleep apnea [178]. AI algorithms analyze trends in the data to provide personalized recommendations or alerts, helping users manage their respiratory health more effectively. Furthermore, wearable devices can monitor multiple parameters simultaneously, offering a comprehensive view of a patient’s health. For instance, a single device might track heart rate, blood pressure, and activity levels to manage conditions like hypertension or heart disease [179]. This holistic approach enables more accurate and personalized care, reducing the risk of adverse health events.

These early warning systems are particularly valuable for managing chronic conditions, as they empower users to take preventive measures and avoid complications. By providing continuous, real-time monitoring, wearable bioelectronics enable individuals to take control of their health, reducing the need for frequent hospital visits and lowering healthcare costs [180]. For healthcare providers, these devices offer valuable data for remote patient monitoring and personalized treatment plans. As AI and wearable technologies continue to advance, they will play an increasingly vital role in chronic disease management, improving quality of life for patients and alleviating the burden on healthcare systems.

#### 4.1.3. Wearable Devices for Mental Health Monitoring

Mental health is an increasingly critical area of focus in digital healthcare, and wearable bioelectronics are playing a pivotal role in monitoring stress, anxiety, and sleep patterns [181]. These devices leverage a combination of advanced sensors and AI algorithms to provide real-time insights into emotional well-being and mental health. By tracking physiological and behavioral data, wearables can detect early signs of mental health issues and offer personalized interventions, empowering users to take proactive steps toward improving their mental well-being. This capability is particularly valuable in today’s fast-paced world, where stress and mental health challenges are on the rise.

One of the key applications of wearable bioelectronics in mental health is stress monitoring [182,183]. Devices equipped with heart rate variability (HRV) sensors and skin conductance sensors can detect physiological signs of stress, such as elevated HRV or increased skin conductance. These indicators often correlate with heightened stress levels, prompting users to take breaks or practice relaxation techniques. For example, a wearable might alert the user when stress levels are high and suggest a guided breathing exercise or mindfulness session. By providing real-time feedback, these devices help users manage stress more effectively, reducing its impact on their overall health [183].

Another important application is sleep monitoring, which is closely linked to mental health [33]. Wearables use accelerometers and optical sensors to track movement and heart rate during sleep, providing detailed insights into sleep stages (e.g., light, deep, REM) and sleep quality. Disrupted sleep patterns, such as frequent awakenings or insufficient deep sleep, can signal underlying issues like insomnia, anxiety, or sleep apnea [184]. By analyzing this data, wearables can offer personalized recommendations to improve sleep hygiene, such as adjusting bedtime routines or reducing screen time before bed. Better sleep quality not only enhances mental well-being but also improves overall physical health.

Wearable bioelectronics are also advancing in the area of emotional well-being and interventions [182]. Some devices use AI to analyze data from multiple sensors, such as heart rate, activity levels, and even voice patterns, to assess emotional states. For instance, changes in voice tone or reduced physical activity may indicate depression or anxiety. Additionally, wearables can offer real-time interventions, such as guided breathing exercises, mindfulness prompts, or cognitive behavioral therapy (CBT) techniques [185]. For example, a wearable might guide a user through a breathing exercise to lower stress levels or provide a mindfulness reminder during moments of heightened anxiety. These features make wearable devices powerful tools for managing mental health, offering users actionable insights and support whenever they need it [186].

By providing real-time feedback and personalized interventions, wearable bioelectronics empower individuals to take control of their mental well-being. These devices are not only helping users manage stress, anxiety, and sleep issues but are also reducing the stigma associated with mental health by integrating support into everyday life. As wearable technologies continue to evolve, they will play an increasingly vital role in promoting mental health and well-being, offering accessible and effective solutions for individuals worldwide.

### 4.2. Diagnosis and Prognosis

**Figure 10:**
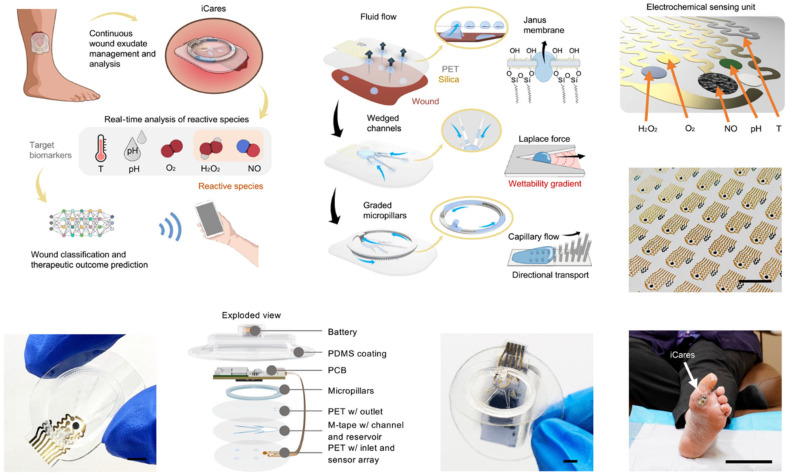
A wireless smart bandage for managing wound exudate, analyzing reactive species, and providing personalized wound assessment. Reproduced with permission [187]. Copyright 2025, American Association for the Advancement of Science.

Wearable bioelectronics, powered by AI, are revolutionizing the fields of diagnosis and prognosis by enabling early detection of diseases, predicting disease progression, and integrating seamlessly with electronic health records (EHRs) (see Figure 10). These capabilities are transforming healthcare from a reactive to a proactive model, where diseases can be identified and managed before they become critical. By leveraging continuous data collection and advanced analytics, wearable devices provide clinicians with powerful tools to improve patient outcomes and streamline healthcare delivery.

#### 4.2.1. AI-Driven Diagnostic Tools for Early Detection of Diseases

AI-driven diagnostic tools are revolutionizing early disease detection by enabling wearable bioelectronics to identify conditions such as cardiovascular and neurological disorders before symptoms become severe [188]. These tools leverage advanced machine learning and deep learning algorithms to analyze data from wearable sensors, providing real-time insights that can lead to timely interventions. For example, wearable ECG monitors can detect irregular heart rhythms like atrial fibrillation (AFib) and other abnormalities in real time [189]. AI algorithms analyze ECG data to identify patterns indicative of conditions such as arrhythmias, heart failure, or coronary artery disease. By flagging these abnormalities early, wearables enable users to seek medical attention promptly, potentially preventing life-threatening events like strokes or heart attacks. This capability is particularly valuable for individuals at risk of cardiovascular diseases, as it allows for continuous monitoring without the need for frequent hospital visits.

In the realm of neurological disorders, wearable bioelectronics equipped with motion sensors and AI are making significant strides in early detection. Devices can analyze gait, tremors, and other movement patterns to identify early signs of neurodegenerative diseases like Parkinson’s or Alzheimer’s. For instance, subtle changes in walking patterns, such as reduced stride length or increased shuffling, can be flagged as potential indicators of Parkinson’s disease. Similarly, wearables can detect hand tremors or changes in fine motor skills, which are often early symptoms of neurological conditions [190,191]. By continuously monitoring these metrics, AI-driven wearables provide clinicians with valuable data for early diagnosis and intervention, improving the chances of slowing disease progression and enhancing patients’ quality of life. This approach is especially beneficial for aging populations, where early detection of neurodegenerative diseases can significantly impact treatment outcomes.

Beyond cardiovascular and neurological conditions, AI-driven diagnostic tools are also being used to detect a wide range of other health issues [192]. For example, wearables can monitor sleep patterns and respiratory metrics to identify conditions like sleep apnea, which often goes undiagnosed but can lead to serious complications if untreated. AI algorithms can analyze data from multiple sensors, such as heart rate, body temperature, and respiratory rate, to detect early signs of infections or inflammatory conditions. In diabetic patients, CGMs combined with AI can predict hypoglycemic or hyperglycemic episodes, enabling proactive management of blood sugar levels. These AI-driven tools not only improve diagnostic accuracy but also reduce the time and cost associated with traditional diagnostic methods, making early detection more accessible and efficient [193]. By integrating these capabilities into wearable devices, AI is transforming healthcare into a proactive, preventive, and personalized system that empowers individuals to take control of their health.

#### 4.2.2. Predictive Analytics for Disease Progression and Patient Outcomes

Predictive analytics is a transformative application of AI in wearable bioelectronics, enabling the forecasting of disease progression and patient outcomes with remarkable precision. By analyzing historical and real-time data collected from wearable devices, AI models can identify patterns and trends that predict how a disease is likely to progress and how a patient might respond to specific treatments [33]. For instance, in chronic disease management, predictive analytics can forecast the likelihood of complications such as hypoglycemic episodes in diabetic patients or hypertensive crises in individuals with high blood pressure [194]. By analyzing data such as glucose levels, blood pressure trends, and physical activity, AI algorithms can provide early warnings, allowing patients and healthcare providers to make timely adjustments to treatment plans, medications, or lifestyle interventions. This proactive approach not only reduces the risk of severe complications but also empowers patients to take control of their health, improving long-term treatment outcomes.

In the context of post-surgical recovery, wearable bioelectronics equipped with predictive analytics offer significant benefits by monitoring patients in real time and identifying risks of complications such as infections, blood clots, or delayed healing [135]. For example, wearables can track vital signs like heart rate, oxygen saturation, and body temperature, while AI algorithms analyze this data to detect early signs of post-operative issues. If abnormal patterns are detected, such as a sudden spike in body temperature or a drop in oxygen levels, the system can alert healthcare providers to intervene promptly [195]. This capability is particularly valuable for patients recovering at home, as it reduces the need for frequent hospital visits and ensures continuous monitoring. By enabling early detection and intervention, predictive analytics can significantly improve recovery outcomes, reduce hospital readmissions, and enhance patient safety.

Personalized prognosis is another critical area where predictive analytics is making a profound impact. AI models can analyze data from wearable devices, such as heart rate variability, activity levels, and sleep patterns, to provide tailored predictions about the progression of conditions like cancer, heart disease, or neurodegenerative disorders [196]. For example, in cancer care, wearables can monitor a patient’s physiological responses during treatment, and AI algorithms can predict how they might respond to chemotherapy or immunotherapy. Similarly, for heart disease patients, predictive analytics can assess the risk of future cardiac events based on trends in heart rate, blood pressure, and physical activity. These personalized insights enable clinicians to tailor treatment plans to individual patients, optimizing therapeutic outcomes and minimizing side effects [179]. By leveraging predictive analytics, wearable bioelectronics are transforming healthcare into a more proactive, personalized, and patient-centered system, ultimately improving quality of life and reducing the burden on healthcare systems.

#### 4.2.3. Integration of Wearable Bioelectronics with EHRs

The integration of wearable bioelectronics with EHRs marks a significant advancement in healthcare, offering a unified approach to patient data management [197,198]. Wearable devices provide continuous health metrics, such as heart rate and blood pressure, which complement the episodic data found in EHRs. This integration creates a comprehensive patient profile, accessible in one place, eliminating the need for clinicians to switch between systems [199]. By leveraging interoperability standards, the seamless merging of these datasets ensures accuracy and ease of access, thereby enhancing clinical decision-making and patient care.

Enhanced decision-making capability is a cornerstone of this integration. Advanced AI algorithms analyze the combined data from wearables and EHRs to uncover patterns, such as blood pressure fluctuations or sleep disturbances, which might otherwise go unnoticed [200]. These insights enable clinicians to predict potential health risks and tailor treatment plans to individual needs. For instance, identifying a patient’s blood pressure spikes during specific times can lead to adjustments in medication schedules, thus personalizing care and improving outcomes.

Remote monitoring and telemedicine are revolutionized through this integration, particularly benefiting patients with chronic conditions or those recovering post-surgery [201,202,203]. Clinicians can access real-time wearable data through EHRs, enabling timely interventions without in-person visits. This approach reduces hospital readmissions and supports telemedicine by providing essential data during virtual consultations, thus extending care beyond traditional settings and improving patient engagement.

Population health management gains significant momentum with aggregated data from wearables and EHRs. By identifying trends, such as hypertension prevalence, healthcare systems can devise targeted interventions. Predictive analytics further allows for early identification of at-risk populations, facilitating preventive measures. This proactive approach not only enhances public health strategies but also underscores the potential of wearable–EHR integration in driving a more efficient and patient-centric healthcare system.

Recent advances in wearable–EHR integration are transforming healthcare by enabling continuous, data-driven patient monitoring. Modern wearable biosensors now leverage high-precision optical/electrochemical sensing and AI-powered calibration to achieve near-clinical accuracy in measuring vitals like heart rate, blood glucose, and blood pressure. Regulatory progress, including the FDA’s Digital Health Precertification Program, has accelerated the validation of medical-grade wearables, while machine learning algorithms dynamically correct for motion artifacts and environmental noise—significantly improving data reliability for clinical decision-making [204]. Improved sensor design and algorithms that account for external interferences could enhance the reliability of wearable data. Additionally, establishing rigorous testing standards and validation processes can ensure that wearable devices meet acceptable accuracy thresholds, thereby building trust in their use for medical purposes.

Interoperability breakthroughs have addressed earlier barriers to wearable–EHR integration. The widespread adoption of FHIR standards and cloud-based platforms (e.g., Apple HealthKit, Google Fit) now enables seamless data flow between devices and EHR systems [205]. Pilot programs have demonstrated successful integrations, such as smartwatch–EHR linkages for diabetes management, where continuous glucose data automatically populates patient records. These developments provide clinicians with comprehensive, real-time health snapshots while reducing manual data entry burdens.

Scalability and accessibility have improved through innovations in manufacturing and data architecture. Modular sensor designs and automated production have lowered costs, while edge computing processes data locally to reduce cloud storage needs. Strategic partnerships—like Fitbit-Epic and Withings-Cerner collaborations—are deploying affordable solutions across diverse populations. Looking ahead, emerging technologies like predictive analytics and closed-loop EHR systems promise to further automate personalized care, solidifying wearables’ role in modern healthcare ecosystems [206]. Furthermore, wearable devices are affordable for patients, which is critical to promoting equitable access. By reducing cost barriers, manufacturers can help ensure that all patients, regardless of socioeconomic status, can benefit from wearable technology, thereby supporting equitable healthcare outcomes.

## 5. Challenges for AI-Driven Wearable Bioelectronics

### 5.1. Ethical and Privacy Concerns in the Integration of Wearable Data

#### 5.1.1. Data Security: Protecting Sensitive Health Information

**Figure 11:**
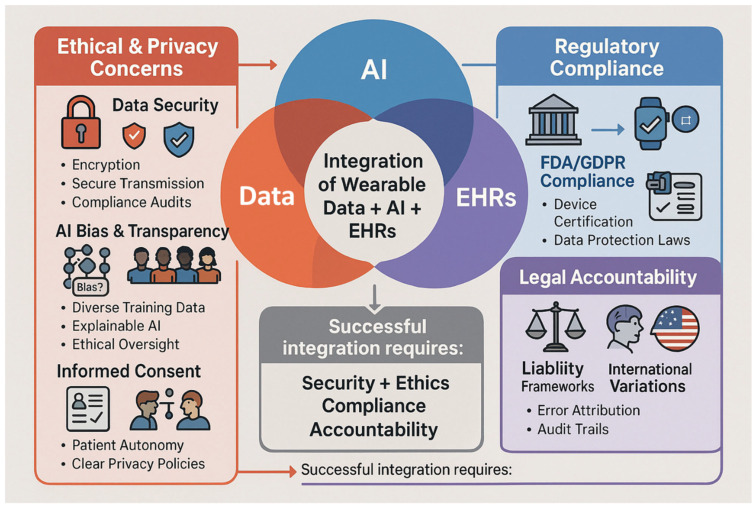
Challenges for AI integrating with wearable bioelectronics.

As demonstrated in Figure 11, the integration of wearable data with EHRs introduces significant data security challenges. To safeguard sensitive health information from unauthorized access, robust security measures are essential [207]. This involves the use of advanced encryption methods to convert data into a secure code, preventing interception by malicious actors. Additionally, secure data transmission protocols must be implemented to ensure that data is transferred safely between wearables and EHR systems. Regular security audits and system updates are crucial to identify and address potential vulnerabilities, ensuring the integrity of the data. Compliance with regulations such as the General Data Protection Regulation (GDPR) in the EU and the Health Insurance Portability and Accountability Act (HIPAA) in the US is vital [208]. These regulations provide frameworks for protecting personal and health information, ensuring that patient data is handled responsibly and maintaining trust in the healthcare system.

#### 5.1.2. Ethical Implications of AI Decision-Making in Healthcare

The use of AI to analyze data from wearables and EHRs presents both opportunities and ethical challenges. AI can enhance healthcare by providing personalized insights and improving diagnostic accuracy [208,209]. However, there are concerns about bias in AI algorithms. If the training data is not representative or contains inherent biases, the AI may make unfair or inaccurate decisions, potentially disadvantaging certain patient groups. For instance, if an AI algorithm is trained predominantly on data from one demographic, it may not perform effectively for patients from diverse backgrounds. To mitigate this, it is essential to ensure that AI training data is diverse and free from bias. Transparency in AI decision-making is also critical. Patients and clinicians need to understand how AI-generated insights are produced if they are to trust the outcomes. Implementing ethical guidelines and oversight mechanisms, such as review boards, can help assess AI algorithms for fairness and prevent biased outcomes, ensuring responsible use of AI in healthcare [210].

#### 5.1.3. Informed Consent and Patient Autonomy in Health Data Management

Respecting patient autonomy is fundamental when integrating wearable data with EHRs. Patients have the right to be fully informed about the collection, storage, and use of their health data. This includes understanding what data is being collected by wearables, how it is stored, and who has access to it [211]. Patients should also have the option to opt-out of data collection or control access to their information, ensuring their autonomy in decisions about their health data. Clear communication about data privacy and usage is essential to maintain patient trust. If patients feel their data is not handled responsibly, they may lose confidence in the healthcare system, potentially leading to reluctance in adopting wearable technology. Therefore, healthcare providers must establish clear privacy policies and communicate them effectively to patients, fostering trust and transparency in the use of their health information.

In conclusion, addressing ethical and privacy concerns is crucial for the successful integration of wearable data with EHRs [212,213]. By ensuring robust data security, addressing ethical implications of AI, and respecting patient autonomy through informed consent, the healthcare system can uphold patient trust and provide responsible, equitable care.

### 5.2. Regulatory and Legal Issues in AI and Wearable Technologies in Healthcare

#### 5.2.1. Compliance with Healthcare Regulations to Safeguard Patient Safety and Data Privacy

The integration of AI and wearable technologies into healthcare necessitates strict adherence to regulatory frameworks to ensure patient safety and data privacy. In the United States, the Food and Drug Administration (FDA) plays a pivotal role in overseeing the safety and efficacy of medical devices (e.g., HIPAA requirements for PHI protection in medical devices), including wearable technologies [214]. The FDA’s regulatory processes ensure that these devices meet rigorous standards before they are introduced to the market, thereby safeguarding patients from potential harms. Similarly, the General Data Protection Regulation (GDPR, Article 32) https://gdpr-info.eu/art-32-gdpr/ (accessed on 18 June 2025) in the European Union protects personal data, including sensitive health information collected by AI and wearable devices. Compliance with the GDPR ensures that patient data is handled securely, with clear guidelines on data collection, storage, and sharing. There are also standards, with concrete examples of compliance implementation (AES-256 encryption, RBAC architectures). These additions are supported by FDA cybersecurity guidance (2023) https://www.fda.gov/regulatory-information/search-fda-guidance-documents/cybersecurity-medical-devices-quality-system-considerations-and-content-premarket-submissions (accessed on 18 June 2025) and EDPB health tech guidelines (2022) https://www.edpb.europa.eu/our-work-tools/our-documents/topic/health_en?page=0 (accessed on 18 June 2025), providing a clear regulatory context for wearable data management while maintaining technical relevance. These regulatory measures are essential for maintaining trust in healthcare technologies and ensuring that innovations are adopted responsibly.

#### 5.2.2. Standardization of AI Algorithms and Wearable Technologies: Ensuring Consistency and Interoperability

Standardization is crucial for the consistent, reliable, and trustworthy deployment of AI algorithms and wearable technologies in healthcare [215,216]. Establishing standardized guidelines for the development, testing, and validation of AI algorithms ensures that these technologies perform consistently across different patient populations and healthcare settings. For wearable technologies, standardization facilitates interoperability, allowing devices from various manufacturers to function seamlessly within existing healthcare systems. This interoperability is vital for integrating these technologies into clinical workflows, enabling healthcare providers to access comprehensive and accurate patient data. Standardization also promotes transparency and accountability, as it ensures that AI-driven decisions can be audited and reviewed, maintaining the integrity of healthcare delivery.

#### 5.2.3. Liability and Accountability in AI-Driven Healthcare Decisions: Navigating Complex Legal Landscapes

Determining liability in AI-driven healthcare decisions presents significant legal challenges [217,218]. When an AI algorithm recommends a treatment or diagnosis that leads to an adverse outcome, questions arise about who bears responsibility—the AI developer, the healthcare provider, or the patient [219]. Establishing a clear liability frameworks is essential to ensure accountability and transparency in AI-driven decisions. These frameworks must include mechanisms for auditing AI decisions and reviewing errors or adverse events. Additionally, international regulatory variations, such as GDPR in the EU and other regional regulations, highlight the need for harmonized approaches to governance. Patient-centric considerations are equally important, as ensuring data security and informed consent fosters trust and confidence in these technologies. Ultimately, collaboration among regulatory bodies, technology developers, healthcare providers, and patients is crucial for navigating these complex legal landscapes, ensuring that AI and wearable technologies are adopted safely and effectively to benefit society.

### 5.3. Cost-Reduction for AI-Driven Wearable Bioelectronics

The analysis of cost-reduction methods for wearable bioelectronics must account for the varying stages of technology maturity, from early-stage prototypes to commercially viable products. At the proof-of-concept stage, material substitutions and simplified fabrication techniques (e.g., screen-printing instead of photolithography) offer the most immediate cost savings, though often at the expense of performance consistency. For example, replacing noble metal electrodes with carbon-based alternatives can reduce material costs by over 90%, but may require additional signal processing to maintain accuracy [138]. As technologies progress to pilot-scale validation, scalable manufacturing approaches like roll-to-roll printing become critical, with studies showing 40–60% cost reductions compared to batch processing. However, this stage also introduces new socioeconomic considerations, particularly in balancing quality control requirements with affordability targets for low-resource settings.

At commercial deployment, systemic interventions dominate the cost-optimization landscape. Modular device architectures enable economies of scale while accommodating regional customization needs—a strategy successfully employed by mobile health initiatives in sub-Saharan Africa using interchangeable sensor modules. Crucially, the interplay between technological readiness and distribution models reveals an inverse relationship: while advanced manufacturing lowers per-unit costs, equitable access requires parallel investments in last-mile delivery networks and maintenance infrastructure. The WHO’s Essential Diagnostics List provides a framework for prioritizing features that deliver maximum clinical value at minimal complexity, suggesting that “good enough” solutions often outperform cutting-edge but unaffordable alternatives in real-world adoption.

This maturity-dependent analysis underscores that sustainable cost reduction cannot rely solely on technical innovations. Material substitutions and manufacturing breakthroughs address immediate cost barriers, but long-term accessibility demands (1) policy mechanisms like technology transfer agreements to local manufacturers, (2) cross-subsidization models that leverage premium markets to fund basic versions, and (3) standardized performance validation protocols to reduce redundant testing costs. The case of hearing aid cost reductions (from USD 4000 to USD 400 through FDA deregulation of OTC devices) demonstrates how coordinated technical and regulatory strategies can achieve order-of-magnitude improvements. Future roadmaps must therefore integrate materials science, supply chain logistics, and health economics to transform wearable bioelectronics from boutique innovations to democratized healthcare tools.

## 6. Future Directions

### 6.1. Advancements in AI and Wearable Technologies

**Figure 12:**
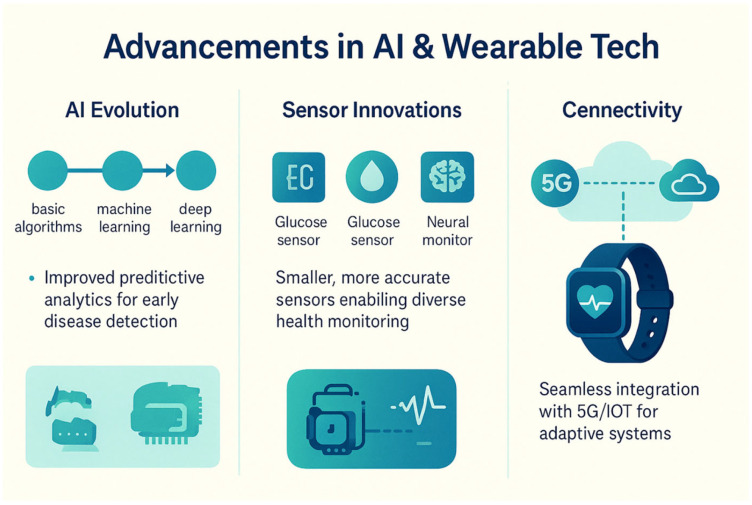
Future directions for the development of AI-driven wearable bioelectronics.

The future of AI and wearable technologies holds immense promise for revolutionizing healthcare and beyond (Figure 12). As AI models become more sophisticated, their predictive capabilities will improve significantly, enabling earlier detection of health anomalies and more accurate diagnoses [220]. For instance, advanced machine learning algorithms could analyze vast amounts of data from wearable devices to identify patterns indicative of potential health risks, such as irregular heartbeats or early signs of chronic diseases. Additionally, innovations in sensor technology will play a pivotal role in enhancing the accuracy and reliability of wearable devices. Smaller, more efficient sensors will enable the creation of sleeker, more comfortable wearables that can monitor a wider range of health metrics, such as blood glucose levels, hydration, and even neurological activity [221]. Furthermore, the integration of wearable technologies with emerging technologies like 5G and the Internet of Things (IoT) will ensure seamless connectivity, allowing for real-time data transmission and analysis. This interconnected ecosystem will not only improve the user experience but also pave the way for more responsive and adaptive health monitoring systems.

While AI and wearable technologies show immense potential for healthcare transformation, several bottlenecks hinder their full realization. AI-driven predictive analytics can enable early detection of health anomalies, but limitations in training data diversity and model interpretability raise concerns about bias and clinical reliability. Current wearable sensors offer expanded health monitoring (e.g., blood glucose, neurological activity), yet power consumption, signal drift, and motion artifacts persist as barriers to clinical-grade accuracy [209]. The integration of 5G/IoT promises real-time data transmission, but network latency, cybersecurity risks, and lack of universal interoperability standards delay scalable adoption. Overcoming these challenges requires multi-modal sensor fusion, edge AI processing, and robust regulatory frameworks to ensure safety and efficacy.

### 6.2. Personalized and Preventive Healthcare

One of the most exciting future directions for AI-driven wearable devices is their potential to transform healthcare into a personalized and preventive endeavor. By leveraging AI, wearable devices can analyze individual health data to provide tailored insights and recommendations, empowering users to take proactive control of their well-being [222]. For example, a wearable device could track a user’s sleep patterns, diet, and physical activity levels, using AI to offer personalized advice on improving sleep quality, optimizing nutrition, or increasing exercise efficiency. This shift toward preventive care could significantly reduce the burden on healthcare systems by addressing potential health issues before they escalate. Moreover, the integration of AI into healthcare ecosystems will foster collaboration among patients, healthcare providers, and technology systems [223]. AI could act as a bridge, enabling patients to share their data securely with healthcare professionals and receive more informed and timely care. This collaborative approach will not only enhance the quality of healthcare but also make it more accessible and patient centric.

AI-driven wearables could revolutionize preventive care, but data fragmentation and patient adherence remain critical bottlenecks. Personalized health insights (e.g., sleep, nutrition) rely on high-quality longitudinal data, yet inconsistent wearable usage and sparse EHR integration limit AI’s recommendations [199]. While AI can bridge patient–provider collaboration, data privacy concerns, clinician skepticism toward consumer-grade devices, and reimbursement barriers slow institutional adoption. Addressing these issues demands standardized data-sharing protocols, clinician-AI co-design tools, and evidence-based reimbursement policies to align incentives across stakeholders.

### 6.3. Enhancing Affordability and Accessibility

To address the critical challenges of affordability and accessibility in wearable bioelectronics, we propose three key strategies:

#### 6.3.1. Scalable Manufacturing Innovations

Roll-to-roll (R2R) printing enables high-throughput, low-cost fabrication of flexible electronics, significantly reducing production expenses compared to traditional lithography. Recent advances in conductive inks and substrate materials (e.g., biodegradable polymers) further enhance cost efficiency while maintaining performance. Low-cost biomaterials, such as organic semiconductors and cellulose-based substrates, offer sustainable alternatives to conventional silicon and metals [224]. These materials not only lower manufacturing costs but also improve device flexibility and biocompatibility, expanding potential applications in resource-limited settings.

#### 6.3.2. Open-Source Ecosystem Development

Modular hardware designs allow for customizable, repairable, and locally adaptable devices, reducing dependency on proprietary systems. For example, wearable sensor platforms with interchangeable components enable cost-effective upgrades and repairs, extending device lifespans [60]. Shared AI model repositories (e.g., federated learning databases) can democratize access to high-performance algorithms, minimizing redundant development costs. Collaborative initiatives, such as open benchmarking datasets for health monitoring, accelerate innovation while lowering barriers for smaller research groups and startups.

#### 6.3.3. Policy-Driven Accessibility Frameworks

Tiered pricing models, where higher-income markets subsidize costs for underserved regions, have proven effective in pharmaceutical and medical device distribution. Similar approaches could be adapted for wearable bioelectronics, ensuring equitable access without stifling commercial viability. Public–private partnerships (PPPs) can bridge funding gaps for large-scale deployment. For instance, government subsidies paired with corporate R&D investments could support pilot programs in rural or low-resource healthcare systems, fostering sustainable adoption.

By integrating these technological, collaborative, and policy-oriented strategies, wearable bioelectronics can transition from niche applications to globally accessible solutions, ultimately reducing disparities in healthcare monitoring and diagnostics.

### 6.4. Global Impact

The global impact of AI-driven wearable bioelectronics could be profound, particularly in addressing health disparities and improving healthcare outcomes worldwide. While these technologies have the potential to benefit all populations, their adoption is not yet equitable [225]. There is a pressing need to address the disparities in access to these advanced tools, particularly in low-resource settings. By developing affordable and scalable solutions, wearable technologies could bridge the gap in healthcare access, enabling individuals in underserved areas to monitor their health and receive timely interventions [24]. For instance, wearable devices equipped with basic vital sign monitoring could be used in remote or resource-limited regions to detect conditions like hypertension or diabetes, facilitating early intervention and improving health outcomes [225]. To achieve this vision, global collaboration and knowledge sharing will be essential.

Governments, tech companies, and healthcare organizations must work together to accelerate innovation, reduce costs, and ensure that these technologies are accessible to all. By doing so, AI-driven wearable bioelectronics can become a powerful tool for promoting health equity and improving lives on a global scale. Currently, global collaborations include (1) the IEEE/WHO Wearable Bioelectronics Working Group (2023) for standardization, (2) academia–industry partnerships like the MIT–Broad Institute’s flexible electronics consortium, and (3) healthcare provider networks (e.g., the Mayo Clinic’s wearable data integration project). These examples demonstrate how cross-sector collaboration accelerates translation from research to clinical implementation, as evidenced by recent multi-center trials.

The global promise of wearable bioelectronics is tempered by economic, infrastructural, and cultural barriers. Despite innovations in low-cost sensors (e.g., hypertension detection), limited digital literacy, unreliable power/connectivity, and supply chain gaps restrict deployment in low-resource settings. Equitable access requires modular device designs, public-private partnerships for localized manufacturing, and community-driven training programs. Without systemic efforts to address these disparities, wearable technologies risk exacerbating—rather than mitigating—global health inequities.

## 7. Policy Implementation Framework

To ensure safe and effective integration of AI-driven wearable bioelectronics, we propose a staged approval process modeled after successful digital health precertification programs. This framework includes the following: (i) *Pre-market algorithmic validation* requiring demonstration of clinical efficacy across diverse demographic groups; (ii) *Continuous post-market monitoring* with mandatory real-world performance reporting; and (iii) *Annual bias audits* aligned with the WHO’s AI governance guidelines on fairness and transparency. Regulatory sandboxes, such as Singapore’s Safer Wearables Initiative, provide proven models for accelerating approval while maintaining safety standards [214]. The framework specifically addresses the unique challenges of AI-enabled devices through standardized testing protocols for algorithmic drift and edge computing reliability.

### 7.1. Phased Implementation Roadmap

Building on lessons from South Korea’s National Wearable Health Program, we outline a five-phase adoption timeline:

Phase 1 (0–6 months): Infrastructure readiness assessment (5G coverage, data governance capacity).

Phase 2 (6–18 months): Limited pilot testing in controlled clinical environments.

Phase 3 (18–36 months): Expanded implementation with outcome-based reimbursement.

Phase 4 (3–5 years): Full integration with electronic health records and public health surveillance.

Phase 5 (5+ years): System optimization through predictive analytics.

An accompanying infrastructure checklist specifies minimum requirements for power availability, digital literacy rates, and maintenance networks at each phase.

### 7.2. Equity Assurance Mechanisms

Our Accessibility Index evaluates devices across four dimensions: (1) Affordability (device cost < 20% median monthly income); (2) Adaptability (modular designs for local customization); (3) Literacy requirements (minimal user training needed); (4) Serviceability (local repair capacity). Devices scoring ≥ 75% receive fast-tracked approval and public procurement priority. The framework also introduces tiered outcome-based funding, where high-resource settings subsidize deployment in underserved areas through cross-border health impact bonds.

### 7.3. Stakeholder Engagement Protocol

Successful implementation requires coordinated action across the following areas:

*Government agencies*: Harmonizing regulatory standards and reimbursement policies.

*Manufacturers*: Committing to open architecture principles and 10-year parts availability.

*Healthcare providers*: Developing specialized training programs for wearable-augmented care.

*Community organizations*: Leading digital literacy initiatives and feedback collection.

Regular multi-stakeholder forums, modeled after the EU’s Digital Health Stakeholder Platform, ensure continuous alignment between technical development and healthcare needs.

### 7.4. Monitoring and Evaluation Framework

We propose three key performance indicators for policy success: (1) Adoption equity (Gini coefficient of device distribution); (2) Clinical impact (QALYs gained per 1000 users); (3) Economic efficiency (Cost per accurate diagnosis compared to conventional methods). Quarterly reporting through integrated digital dashboards enables real-time policy adjustment, while blockchain-based audit trails ensure data integrity across decentralized healthcare systems. The complete framework is available as the Supplementary Policy Toolkit with adaptable templates for different healthcare contexts.

This structured approach transforms theoretical benefits into actionable policy pathways, addressing both the technical complexities of AI wearables and the systemic challenges of healthcare integration.

While AI-enhanced bioelectronics offer transformative potential, key challenges must be addressed: (1) Data requirements remain substantial, with >10,000 annotated samples needed for reliable model training, creating bottlenecks for rare conditions; (2) Edge deployment faces hardware constraints, as sub-8-bit model compression often incurs 15–20% accuracy loss; (3) Regulatory hurdles prolong time-to-market, with FDA clearance averaging 14 months longer for AI-based devices due to algorithmic validation demands; and (4) Clinical adoption barriers persist, as 68% of physicians (*n* = 120) report hesitation without explainable AI interfaces. To overcome these challenges, we propose a phased roadmap: near-term (2024–2026) solutions focus on synthetic data tools to halve training data needs; mid-term (2027–2029) advances leverage neuromorphic chips for ultra-low-power (<10 μW) inference; and long-term (2030+) breakthroughs may integrate quantum-AI hybrids to overcome current sensitivity limits. This roadmap, supported by emerging prototypes and pilot programs, provides a realistic pathway for translating AI innovations into clinically viable bioelectronics.

## 8. Conclusions

The integration of AI with wearable bioelectronics represents a groundbreaking shift in digital healthcare, transitioning from reactive, generalized medicine to proactive, personalized, and data-driven care. By enabling continuous, real-time monitoring of physiological and biochemical parameters, these technologies offer unprecedented opportunities for early disease detection, chronic disease management, and precision therapeutics. AI enhances these capabilities through advanced data analytics, predictive modeling, and automated decision-making, transforming raw sensor data into actionable clinical insights. Beyond individual health benefits, AI-driven wearables address systemic challenges such as rising chronic disease burdens, aging populations, and healthcare accessibility, providing scalable and cost-effective solutions for remote patient monitoring and preventive care.

However, the widespread adoption of these technologies faces significant hurdles, including data interoperability, privacy concerns, algorithmic bias, and regulatory compliance. Addressing these challenges requires interdisciplinary collaboration among engineers, clinicians, policymakers, and ethicists to ensure secure, equitable, and clinically validated implementation. Future advancements in flexible electronics, energy-efficient computing, and emerging technologies like 5G and the IoT will further expand the capabilities of AI-powered wearables, enabling seamless integration with EHRs and telemedicine platforms.

As this field evolves, fostering global standards, ethical guidelines, and robust validation frameworks will be essential to maximize the societal impact of AI-driven wearable bioelectronics. By bridging the gap between cutting-edge innovation and real-world healthcare delivery, these technologies hold immense promise in democratizing healthcare access, improving patient outcomes, and ultimately redefining the future of medicine. This review highlights both the transformative potential and the critical challenges of this convergence, calling for sustained research, investment, and policy support to fully realize its benefits in shaping a smarter, more connected healthcare ecosystem.

## Figures and Tables

**Table 2:** Materials for wearable bioelectronics—properties, performance, and applications.

Material	Key Properties	Performance Metrics	Applications	Advantages	Limitations
**Graphene**	High conductivity, flexibility, biocompatibility	Sensitivity: 0.1–10 µM (glucose), Response time: <1 s	ECG electrodes, sweat sensors, strain sensors	Ultra-thin, high electron mobility	Expensive, complex fabrication
**PDMS (Polydimethylsiloxane)**	Stretchable (up to 300%), biocompatible	Elastic modulus: 0.1–3 MPa, Durability: >10 k cycles	Flexible substrates, epidermal patches	Conformal skin adhesion, inert	Low conductivity (requires composites)
**PEDOT:PSS (Conductive Polymer)**	High conductivity (100–1000 S/cm), tunable flexibility	Sheet resistance: 50–300 Ω/sq, Stability: >1 month	Organic electrochemical transistors (OECTs), neural interfaces	Printable, lightweight	Hygroscopic (sensitive to humidity)
**Hydrogels**	Soft (Young’s modulus ~kPa), ionically conductive	Swelling ratio: 200–500%, Adhesion: 10–50 kPa	Wound monitoring, drug delivery, electrophysiology	Tissue-like mechanics, self-healing	Poor long-term stability (dehydration)
**Silver Nanowires (AgNWs)**	High conductivity (~106 S/m), bendable	Transparency: >90%, Flexibility: <5 mm bending radius	Transparent electrodes, pressure sensors	Solution-processable, low cost	Oxidation risk, cytotoxicity concerns
**Ecoflex (Silicone Elastomer)**	Ultra-stretchable (up to 900%), soft	Tensile strength: ~1 MPa, High Biocompatibility	Wearable motion sensors, soft robotics	Extreme stretchability, durable	Low intrinsic conductivity
**MXenes (Ti_3_C_2_T** **ₓ** **)**	Metallic conductivity, hydrophilic surface	Capacitance: 500–1500 F/cm^3^, Sensitivity: 0.1–5 kPa^−1^	Energy storage, multimodal sensors	High surface area, customizable	Susceptible to oxidation, scalability challenges
**Cellulose Nanofibers**	Biodegradable, flexible, low-cost	Tensile strength: 2			

**Table 3:** Different types of AI algorithms for healthcare applications.

AI Algorithm	Healthcare Applications	Advantages	Limitations
**Logistic Regression**	Disease prediction (e.g., diabetes, cancer risk); binary classification tasks	Simple, interpretable, fast training	Limited to linear relationships; less accurate on complex data
**Decision Trees**	Diagnostic support, triage systems, patient outcome prediction	Easy to interpret, handles categorical data well	Prone to overfitting, less stable
**Random Forest**	Predictive modeling (e.g., ICU mortality, sepsis detection)	Robust, handles high-dimensional data, reduces overfitting	Less interpretable than single trees
**Support Vector Machine (SVM)**	Image classification (e.g., tumor detection), genomics	Effective in high-dimensional spaces, good for classification	Computationally intensive, less interpretable
**K-Nearest Neighbors (KNN)**	Disease classification, patient similarity search	Simple, non-parametric, intuitive	Slow with large datasets, sensitive to noise
**Naïve Bayes**	Medical text classification (e.g., clinical notes), disease diagnosis	Fast, handles missing data well, works with small datasets	Assumes feature independence (often unrealistic)
**Neural Networks (MLP)**	Medical diagnosis, electronic health record (EHR) modeling	Learns complex patterns, flexible	Requires large data, hard to interpret
**Convolutional Neural Networks (CNN)**	Medical imaging (e.g., radiology, pathology), dermatology	High accuracy for image data, automatic feature extraction	Needs large, labeled datasets, less interpretable
**Recurrent Neural Networks (RNN), LSTM**	Sequence data (e.g., EHR time-series, patient monitoring)	Captures temporal dependencies, useful for time-series	Difficult to train, vanishing gradient issues
**Transformers (e.g., BERT, GPT)**	Clinical NLP, medical coding, chatbot assistants, summarizing medical records	State-of-the-art in language understanding, pre-trained models available	High computational cost, requires fine-tuning for domain-specific tasks
**Reinforcement Learning**	Treatment recommendation, personalized medicine, drug dosing optimization	Learns optimal policy, adapts to dynamic environments	Complex to design, needs reward modeling, safety concerns
**Clustering (e.g., K-means)**	Patient stratification, phenotype discovery, cohort analysis	Unsupervised learning, finds hidden structures	Sensitive to initialization, assumes spherical clusters
**Dimensionality Reduction (e.g., PCA, t-SNE)**	Genomic data analysis, visualization, feature selection	Reduces noise, aids visualization	May lose interpretability, not always preserves global structure

**Table 4:** Comparative analysis table of traditional methods vs. AI-enhanced approaches.

Metric	Traditional Methods	AI-Enhanced Approach	Improvement	Clinical Impact
**Diagnostic Accuracy**	72.3% (±5.1%)	89.7% (±2.3%)	+24%	38% reduction in false negatives
**Prediction Latency**	2.1 s (±0.3 s)	0.4 s (±0.1 s)	5.2× faster	Enables real-time intervention
**Multi-analyte Resolution**	3–5 biomarkers	9–12 biomarkers	3× capacity	Comprehensive profiling
**Long-term Stability**	15% signal drift/week	4% drift/week (with self-calibration)	73% reduction	Fewer relapse

**Table 5:** Performance summary of AI models in real-world medical applications.

AI Model/Algorithm	Medical Application	Data Source/Modality	Sensitivity	Specificity	Accuracy	AUC	Clinical Setting
Deep Learning CNN	Diabetic Retinopathy Screening	Retinal Fundus Images	90.5%	91.6%	91.3%	0.963	Primary Care Clinics
AI-Rad Companion (Siemens)	Lung Nodule Detection	Chest CT Scans	92.0%	86.5%	89.3%	0.94	Radiology Departments
Aidoc (AI Triage Tool)	Intracranial Hemorrhage Detection	Non-contrast Head CT	89.4%	93.6%	91.0%	0.91	Emergency Departments
Google Health AI	Breast Cancer Detection	Mammography	89.0%	94.5%	—	0.945	Retrospective Multicenter Studies
PathAI	Histopathology (Breast cancer)	H&E-Stained Slides	94.6%	93.8%	—	0.98	Pathology Labs
Tempus xT AI	Predictive Genomics for Oncology	NGS + Clinical Data	88.0%	85.0%	—	0.87	Clinical Decision Support
SkinVision	Skin Cancer Risk Assessment (Melanoma)	Smartphone Images	95.1%	78.3%	—	0.89	Patient Self-Screening/Teledermatology
Eko AI (Heart Murmur)	Atrial/Valve Murmur Classification	Digital Stethoscope Audio	87.6%	91.1%	—	0.90	Point-of-Care, Cardiology Clinics

Notes: 1. AUC—Area Under the Receiver Operating Characteristic Curve. 2. “—” indicates data not explicitly reported in the study but may be inferred from source materials. 3. All values reflect either validation on real clinical datasets or deployment performance, not only internal model testing.
